# *Phytoene synthase 2* can compensate for the absence of *PSY1* in the control of color in *Capsicum* fruit

**DOI:** 10.1093/jxb/eraa155

**Published:** 2020-03-27

**Authors:** So-Jeong Jang, Hyo-Bong Jeong, Ayoung Jung, Min-Young Kang, Suna Kim, Sun-Hwa Ha, Jin-Kyung Kwon, Byoung-Cheorl Kang

**Affiliations:** 1 Department of Plant Science, Plant Genomics & Breeding Institute, and Research Institute of Agriculture and Life Sciences, Seoul National University, Gwanak-gu, Seoul, Republic of Korea; 2 Food and Nutrition in Home Economics, Korea National Open University, Jongno-Gu, Seoul, Republic of Korea; 3 Department of Genetic Engineering and Graduate School of Biotechnology, College of Life Sciences, Kyung Hee University, Giheung-gu, Yongin-si, Gyeonggi-do, Republic of Korea; 4 Fondazione Edmund Mach, Italy

**Keywords:** Capsanthin-capsorubin synthase, *Capsicum*, carotenoid, color complementation, mature fruit color, pepper, phytoene synthase, VIGS, virus-induced gene silencing

## Abstract

*Phytoene synthase 1* (*PSY1*) and *capsanthin-capsorubin synthase* (*CCS*) are two major genes responsible for fruit color variation in pepper (*Capsicum* spp.). However, the role of *PSY2* remains unknown. We used a systemic approach to examine the genetic factors responsible for the yellow fruit color of *C. annuum* ‘MicroPep Yellow’ (MY) and to determine the role of *PSY2* in fruit color. We detected complete deletion of *PSY1* and a retrotransposon insertion in *CCS*. Despite the loss of *PSY1* and *CCS* function, both MY and mutant F_2_ plants from a cross between MY and the ‘MicroPep Red’ (MR) accumulated basal levels of carotenoids, indicating that other *PSY* genes may complement the loss of *PSY1*. qRT-PCR analysis indicated that *PSY2* was constitutively expressed in both MR and MY fruits, and a color complementation assay using *Escherichia coli* revealed that PSY2 was capable of biosynthesizing a carotenoid. Virus-induced gene silencing of *PSY2* in MY resulted in white fruits. These findings indicate that PSY2 can compensate for the absence of PSY1 in pepper fruit, resulting in the yellow color of MY fruits.

## Teaser

*PSY2* can compensate for the absence of *PSY1* in yellow pepper fruit, allowing a basal level of carotenoid accumulation to occur, and hence it should be considered alongside *PSY1* and *CCS* as an essential gene in the control of fruit coloration.

## Introduction

Peppers (*Capsicum* spp.) are one of the most popular fruits in the world. Among their traits, color is one of the most important for aesthetic value and in relation to nutritional benefits. The distinct colors of mature fruit result from the conversion of chloroplasts to chromoplasts during ripening. Since chromoplasts possess a higher capacity to biosynthesize and sequester carotenoids than chloroplasts, mature pepper fruit often display ivory-to-red colors depending on chromoplast development and carotenoid biosynthesis (Sun *et al.*, 2018). Carotenoids belong to a group of tetraterpenoids derived from eight isoprene units, thus contain 40 carbons in their polyene backbone (Jackson *et al.*, 2008). Carotenoids are essential for human nutrition and health as they provide a dietary source of provitamin A and serve as antioxidants that can reduce the incidence of many diseases, including cardiovascular diseases and cancers (Fraser and Bramley, 2004). Carotenoids also play essential roles in plants, functioning in photosynthesis and photo-protection, and providing precursors for phytohormones such as abscisic acid and strigolactones (Nambara and Marion-Poll, 2005; Domonkos *et al.*, 2013; Al-Babili and Bouwmeester, 2015).

Carotenoid biosynthesis begins with the formation of phytoene, which is catalysed by phytoene synthase (PSY). There are at least two *PSY* genes in plants, except for *Arabidopsis thaliana*, which only has a single *PSY* gene. Multiple *PSY* genes in one plant can explain different carotenogenesis in various tissues; for example, in tomato (*Solanum lycopersicum*), *PSY1* functions in the fruit, *PSY2* in the leaves, and *PSY3* in the roots (Fantini *et al.*, 2013, Wang *et al.*, 2014; Yuan *et al.*, 2015). Following a series of desaturation and isomerization steps, phytoene is converted into lycopene, which is the major carotenoid component in mature tomato fruit. By contrast, the red color of pepper fruit is caused by the accumulation of capsanthin and capsorubin, which are also derived from lycopene (Paran and van der Knaap, 2007). This pepper-specific process is regulated by capsanthin-capsorubin synthase (CCS), which converts antheraxanthin and violaxanthin into capsanthin and capsorubin, respectively (Bouvier *et al.*, 1994).

The three-locus model (*C1*, *C2*, and *Y*) has been proposed to explain the color variation in pepper fruit (Hurtado-Hernandez and Smith, 1985). In this model, mature fruit colors can be classified into eight groups according to the allelic combinations of three genes, from white (*c1 c2 y*) to red (*C1 C2 Y*). Analyses of candidate carotenoid biosynthetic genes have indicated that the *C2* and *Y* loci encode *PSY1* and *CCS*, respectively (Lefebvre *et al.*, 1998; Popovsky and Paran, 2000; Huh *et al.*, 2001); however, the third locus, *C1*, is yet to be characterized. Since the identification of the genes encoded by the *C2* and *Y* loci, many studies have explored the color variation in pepper fruit based on the allelic variations of *PSY* and *CCS*. In general, peppers with yellow fruit have structural mutations in the coding region of *CCS* that lead to a premature stop codon (Lefebvre *et al.*, 1998; Popovsky and Paran 2000; Ha *et al.*, 2007; Li *et al.*, 2013). The early translational termination of *CCS* can also result in orange fruit, as has been observed in *C. annuum* ‘Fogo’ (Guzman *et al.*, 2010). By contrast, Kim *et al.* (2010) found that a splicing mutation in *PSY1* impairs the activity of its enzyme product and leads to orange bonnet pepper fruit (*C. chinense* ‘Habanero’).However, allelic variations of *PSY* and *CCS* cannot fully explain the differences in fruit color across all pepper accessions (Jeong *et al.*, 2019). Therefore, identification of the last color-determining gene, *C1*, is necessary to complete the picture.

Studies aimed at identifying *C1* and validating the three-locus model for fruit color variation in pepper have investigated the roles of other carotenoid biosynthetic genes. Analysis of capsanthin biosynthetic genes using virus-induced gene silencing (VIGS) in detached red pepper fruit has shown that, in addition to *PSY1* and *CCS*, *lycopene ß-cyclase* (*Lcyb)* and ß*-carotene hydroxylase (CrtZ-2)* are also required for the formation of the normal red color (Tian *et al.*, 2014). In addition, Borovsky *et al.* (2013) reported that an ethyl methanesulfonate (EMS)-induced nucleotide substitution in the *CrtZ-2* coding region led to the conversion of red fruit into orange in *C. annuum* ‘Maor’.However, natural mutations of these genes have not been identified, and therefore they are not candidates for the *C1* locus.

The carotenoid biosynthetic pathways and the functions of the genes involved have been elucidated using strains of *Escherichia coli* as heterologous hosts in color complementation assays (Perry *et al.*, 1986; Cunningham and Gantt, 2007). The pAC-BETA vector contains the *crtE*, *crtB*, *crtI*, and *crtY* carotenogenic genes from *Erwinia herbicola*, which can produce *β*-carotene when transformed into *E. coli* (Cunningham *et al.*, 1996). By contrast, the pAC-85b vector does not contain *crtB*, a homolog of *PSY*, and when expressed in *E. coli*, *β*-carotene can only be produced when complemented with a gene encoding a PSY enzyme (Cunningham and Gantt, 2007). Through complementation assays using pAC-85b, the function of the *PSY1* transcriptional variants of both barley (*Hordeum vulgar*e) and tomato has been revealed (Rodríguez-Suárez *et al.*, 2011; Kang *et al.*, 2014), and the catalytic activities of multiple PSY enzymes have also been analysed in loquat (*Eriobotrya japonica*) (Fu *et al.*, 2014). Moreover, the role of *Lcyb* in fruit flesh color has been confirmed in papaya (*Carica papaya*) by using a color-complementation test with the pAC-LYC construct, which contains genes for lycopene production (Blas *et al.*, 2010). A wide range of carotenoid-related enzymes have been analysed using this approach, such as the carotenoid cleavage dioxygenases (Kim *et al.*, 2013); however, *PSY* genes of pepper have not yet been examined.

In this study, we examined seven carotenoid biosynthetic genes in order to determine the genetic factors that regulate the yellow fruit color in *C. annuum* ‘MicroPep Yellow’ (MY). Among them, we identified structural mutations in *PSY1* and *CCS*. Despite the associated functional knockout of PSY1, which catalyses the rate-limiting step of carotenoid biosynthesis, basal levels of carotenoids were still detected in MY using ultra-performance liquid chromatography analysis. We postulated that *PSY2* may contribute to the formation of the yellow color in MY fruit, and to test this, we conducted qRT-PCR analysis of *PSY2* expression and together with color complementation assays. VIGS of *PSY2* was used to confirm the function of this gene in MY. The results showed that *PSY2* is expressed during all stages of fruit development and it is a functionally active gene. We suggest that PSY2 can compensate for the absence of PSY1 in pepper fruit.

## Materials and methods

### Plant material

**Fig. 1.**
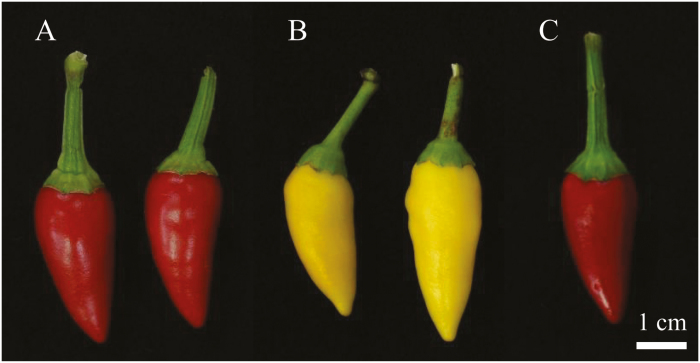
Mature pepper fruits of the genotypes used in this study. (A) ‘MicroPep Red’ (MR), (B) ‘MicroPep Yellow’ (MY), and (C) a F_1_ hybrid derived from the MR × MY cross.

The *Capsicum annuum* accessions ‘MicroPep Red (MR)’ and ‘MicroPep Yellow (MY)’ were selected from the germplasm of the Horticultural Crops Breeding and Genetics laboratory (Seoul National University, Republic of Korea). The mature fruit of MR and MY were red and yellow, respectively (Fig. 1). A total of 281 F_2_ individuals were obtained from a cross between MR and MY and grown at the greenhouse of Seoul National University (Suwon, Republic of Korea).

### Extraction of DNA and RNA

Genomic DNA (gDNA) was extracted from the young leaves of F_2_ individuals from a cross between MR and MY, and from the parental lines, using a modified cetyltrimethylammonium bromide (CTAB) method (Porebski *et al.*, 1997). The leaf tissues were homogenized using 3-mm steel beads with TissueLyserII (Qiagen). The gDNA concentration were measured and diluted to 20 ng μl^–1^ for further experiments.

Total RNA was extracted from target tissues, immediately frozen in liquid nitrogen, and ground into a fine powder. Fruit pericarps were sampled for RNA extraction at three different developmental stages: immature stage (S1), breaker stage (S2), and mature stage (S3). The RNA was extracted using a MG RNAzol kit (MGmed, Seoul, Republic of Korea), following the manufacturer’s instructions. The cDNA was synthesized from 2 μg RNA using an EasyScript Reverse Transcriptase kit (TransGen, Beijing, China) with oligo(dT) primers. The resulting cDNAs were used for the expression analyses.

### Candidate gene analysis

Reference sequences of seven carotenogenic genes in pepper were obtained from the Sol Genomics Network (https://solgenomics.net/). *PDS* was not included as its mutation results in distinct effects. The pepper *PSY2* and *PSY3* sequences have not previously been identified; therefore, the tomato *PSY2* and *PSY3* sequences were BLAST-searched against the pepper genome (CM334 v.1.55). As a result, we obtained the coding sequences for *CA02g20350* (92% sequence identity with tomato *PSY2*; *Solyc02g081330*) and *CA01g12040* (93% sequence identity with tomato *PSY3*; *Solyc01g005940*). Primers were designed to amplify the full length of these candidate genes (Supplementary Table S1 at *JXB* online), and PCR was performed using PrimeSTAR GXL DNA polymerase (Takara Bio). The resulting amplicons were separated on a 1% agarose gel and the DNA was recovered using a LaboPass PCR clean-up kit (Cosmo Genetech, Seoul, Republic of Korea). Sanger sequencing was performed at Macrogen (Seoul, Republic of Korea). The nucleotide sequences were analysed using the DNASTAR Lasergene SeqMan program. The carotenoid biosynthetic pathway of pepper is shown in Supplementary Fig. S1.

### Genome walking from a gene adjacent to *PSY1*

To examine the structural variation of *PSY1* in MY, genome walking was performed starting from an adjacent gene. Separate aliquots of MY gDNA were digested using four restriction enzymes, *Dra*I, *Eco*RV, *Pvu*II, and *Stu*I. Each digested DNA was separately ligated to a genome walker adaptor of a Universal GenomeWalker 2.0 kit (Takara Bio). After the library construction, consecutive PCR amplifications were performed. For the primary PCR, an outer adaptor primer (AP1) and an outer gene-specific primer (GSP1) were used. The primary PCR product was diluted 50× and used as a template for the secondary PCR with a nested adaptor primer (AP2) and a nested gene-specific primer (GSP2). *Capana04g002520* is located in the adjacent 5′ region of *PSY1* (*Capana04g002519*) based on the pepper ‘Zunla’ reference genome (v.2.0, Sol Genomics Network), so gene-specific primers were first designed to target its coding region. The secondary PCR amplicons were purified and sequenced by Macrogen. The next set of gene-specific primers was subsequently designed based on the sequenced genomic region. The primary PCR conditions were as follows: eight cycles of 94 °C for 25 s and 72 °C for 3 min, then 32 cycles of 94 °C for 25 s and 67 °C for 3 min. The secondary PCR conditions were as follows: six cycles of 94 °C for 25 s and 72 °C for 3 min, then 19 cycles of 94 °C for 25 s and 67 °C for 3 min.

### Long-range PCR amplification of *CCS*

To investigate whether an insertion was present in MY *CCS*, a long-range PCR was performed using primers for CCS amplification (Supplementary Table S1) and PrimeSTAR GXL DNA polymerase (Takara Bio). A two-step PCR was performed for 30 cycles of 98 °C for 10 s and 68 °C for 10 min. The resulting amplicons were purified and cloned into a modified T-blunt vector (SolGent, Daejeon, Republic of Korea). The plasmid DNA was extracted using an AccuPrep plasmid mini extraction kit (Bioneer, Daejeon, Republic of Korea) and sequenced by Macrogen.

### Development of a sequence-characterized amplified region marker for the structural variations of *PSY1* and *CCS*

Sequence-characterized amplified region (SCAR) markers for *PSY1* and *CCS* were designed based on the identified structural variations. A common (COM) primer and an allele-specific primer for the MR and MY genotypes were paired for gene amplification (Supplementary Table S2). The SCAR markers were used to genotype the MY × MR F_2_ plants, and to screen germplasm accessions of interest in cases where *PSY1* had not been amplified in a previous study (Jeong *et al.*, 2019). The SCAR marker PCR conditions were as follows: 35 cycles of 98 °C for 10 s, 60 °C for 30 s, and 72 °C for 1 min. The resulting amplicons were separated on a 2% agarose gel.

### Expression analysis of *PSY2* using quantitative real-time PCR

For measurement of *PSY2* expression, quantitative real-time PCR (qRT-PCR) was carried out using a LightCycler 480 system (Roche) using SYTO 9 stain (ThermoFisher Scientific). The reaction mixture comprised a total volume of 20 μl, containing 2 μl 4× diluted cDNA, 0.3 μl R Taq (Takara Bio), 2 μl 10× PCR buffer, 2 μl 10 mM dNTPs, 0.5 μl 10 pmol primers, and 0.5 μl SYTO 9. The primer sequences were as follows: 5′-AGACAGAGGTGGAATTTTGGGTCT-3′ (forward) and 5′-CAAATTCCCCGGAAGCACA-3′ (reverse). The real-time PCR involved 45 cycles of 95 °C for 10 s, 60 °C for 20 s, and 72 °C for 20 s. Three replicate reactions were performed. *Actin* served as an internal control.

### Complementation assays for *PSY2* in *E. coli*

To examine the enzymatic activity of PSY2, a complementation assays conducted using pAC-85b (Addgene plasmid #53282). pAC-85b is derived from pAC-BETA (Addgene plasmid #53272), which contains *CrtE*, *CrtY*, *CrtI*, and *CrtB* (the carotenogenic gene cluster) from *Erwinia herbicola* for *β*-carotene production. pAC-85b lacks the *PSY* homolog *CrtB* compared to pAC-BETA, meaning that *E. coli* transformed with pAC-85b can only produce β-carotene when complemented with a gene encoding a PSY.

For the transgenic expression of *PSY1* and *PSY2*, pET-28a(+) (Novagen; Merck Millipore) was used (Supplementary Fig. S2). To construct pET-PSY1 and pET-PSY2, the full-length cDNA fragments were amplified using the following primers with a 4-bp adapter and 6-bp restriction site (in italics): 5′-CACA*GGATCC*ATGTCTGTTGCCTTGTTATGG-3′ (*PSY1* forward) and 5′-CACA*GTCGAC*ATCCTGATTTCATGTTCTTGTAGAAG-3′ (*PSY1* reverse); 5′-CCAA*GGATCC*ATGTCTGTTGCTTTGTTGTGG-3′ (*PSY2* forward) and 5′-GGAA*GTCGAC*CAACTTCATTCATGTCTTTGTTAGTG-3′ (*PSY2* reverse). The amplicons were cloned into the *Bam*HI and *Sal*I sites of pET-28a(+). After the sequences were confirmed, pAC-85b and either pET-PSY1 or pET-PSY2 were co-transformed into the *E. coli* BL21(DE3) competent cells (TransGen), and named PC1 and EXP respectively. The control cells were co-transformed with pAC-85b and pET-28a(+) for the negative control (NC), and with pAC-BETA for the positive control (PC2) for a visual confirmation of the *β*-carotene content.

The transformed *E. coli* cells were grown overnight at 28 °C in 2 ml of a Luria-Bertani (LB) liquid medium containing 50 mg ml^–1^ kanamycin and 35 mg ml^–1^ chloramphenicol and used to inoculate 10 ml LB medium containing the same antibiotics. The cells were grown at 28 °C to OD_600_=0.5–0.7, then 50 μM isopropylthio-β-galactoside (IPTG) was added and the cells were further incubated in darkness at 16 °C for 24 h. Cell pellets were harvested by centrifugation and the colors of the pellets were analysed.

### Immunoblot assays of the PSY proteins produced by transgenic *E. coli*

NC, EXP, and PC1 *E. coli* were grown at 16 °C for 24 h, after which 2 ml of each sample was transferred into 1.5-ml tubes, harvested by centrifugation, and frozen at –20 °C. The frozen cells were resuspended in 800 μl distilled water and 200 μl 5× sample buffer, then incubated at 98 °C for 5 min for denaturation. The proteins were separated using SDS-PAGE at 130 V for 1.5 h. After the electrophoresis, one repeat was cut from the gel and transferred to an Amersham Hybond^TM^ -P membrane (GE Healthcare), following the manufacturer’s instructions. The other sample replicate was stained with Coomassie Brilliant Blue solution.

The membrane was blocked using TBST buffer containing 5% BSA for 1 h at room temperature. An antibody was raised against the middle region of tomato PSY1 in mouse [anti-SlPSY1 (M)] as a primary antibody to bind to PSY2 for amino acid sequence similarity between SlPSY1 and CaPSY2. Anti-mouse-IgG-HRP was used as a secondary antibody. The anti-PSY1 (M) antibody (1 mg μl^–1^) was diluted 5000:1 using TBST buffer containing 5% BSA. The primary antibodies were applied to the blocked membrane, and incubated at overnight at room temperature. The membranes were washed twice by shaking and twice on a rocker at medium speed for 10 min with TBST buffer. The secondary antibody (1 mg μl^–1^) was diluted 5000:1 with TBST buffer, and applied to the membrane for 3 h on rocker at gentle speed. The membrane was washed as described above. Clarity^TM^ Western ECL substrate (Bio-Rad) and Fusion FX (Vilber Lourmat, Collegien, France) were used for signal development and detection following the manufacturers’ instructions. The expected PSY2 sizes were calculated using a protein molecular weight calculator (www.sciencegateway.org/tools/proteinmw.htm).

### Carotenoid extraction and ultra-performance liquid chromatography analysis

Carotenoids were extracted using methods specific to the material being examined. For the pericarp tissues, the extraction and saponification were conducted as described by Kim *et al.* (2016). For the *E. coli* transformants, cell pellets were obtained using centrifugation, from which the carotenoids were extracted with 600 μl HPLC-grade acetone (Honeywell, Charlotte, USA), as described by López-Emparán *et al.* (2014).

For the extracts derived from both the pericarps and *E. coli* cell pellets, the carotenoids were analysed using ultra-performance liquid chromatography (UPLC) on an Acquity UPLC-H-Class system (Waters). The compounds were separated using an Acquity UPLC HSS T3 column (2.1×100, 1.8 μm) at 35 °C. The mobile phase was a binary solvent system consisting of phase A (acetonitrile/methanol/methylene chloride, 65/25/10, v/v/v) and phase B (distilled water). The gradients were programmed according to the method described by Kim *et al.* (2016). For the qualitative and quantitative analyses, 11 carotenoid standards were purchased from Sigma-Aldrich, namely antheraxanthin, capsanthin, capsorubin, lutein, neoxanthin, violaxanthin, zeaxanthin, *α*-carotene, *α*-cryptoxanthin, *β*-carotene, and *β*-cryptoxanthin.

### VIGS of *PSY2* in the MY accession

Ligation-independent cloning (LIC) was conducted for the construction of pTRV2-PSY2 as described by Kim *et al.* (2017). The partial coding sequence of *PSY2* was amplified with the following primers containing 15-bp adapter sequences (in italics): 5′- *CGACGACAAGACCCT*TGTTGCTTTGTTGTGGGTTG-3′ (forward) and 5′- *GAGGAGAAGAGCCCT*TCCCAAGTCCGAATATCTCAA-3′ (reverse). The purified PCR product and *Pst*I-digested pTRV2-LIC vector were both treated with T4 DNA polymerase (Enzymatics, Beverly, USA) and 5× blue buffer, and with 10 mM dATP or dTTP, respectively. Both mixtures were incubated at 22 °C for 30 min, and then 75 °C for 20 min. A total of 30 ng PCR product and 400 ng TRV2-LIC vector were then mixed and incubated at room temperature for 15 min. The mixture was transformed into *E. coli* DH5α competent cells (TransGen). Plasmids were extracted, sequenced by Macrogen, and introduced into *Agrobacterium tumefaciens* strain GV3101 through electroporation at 2.0 kV. For the negative VIGS control, pTRV2-GFP was used. Both the pTRV2-LIC and pTRV2-GFP vectors were kindly provided by Dr Doil Choi of Seoul National University.

*Agrobacterium* carrying pTRV1, pTRV2-GFP, or pTRV2-PSY2 were grown overnight at 30 °C in LB medium with 50 μg ml^–1^ rifampicin and kanamycin, and then harvested using centrifugation. The cells were resuspended in 10 mM MES, 10 mM MgCl_2_, and 200 μM acetosyringone to a final OD_600_ of 0.7–0.8, and incubated in a rocking incubator at room temperature for 3 h. Cell cultures containing pTRV1 and either of the pTRV2 constructs were mixed at a 1:1 ratio and infiltrated into the abaxial side of both pepper cotyledons. The inoculated plants were incubated at 16 °C in the dark for 1 d, then grown at 25 °C under a16/8-h light/dark photoperiod.

### Expression analysis of *PSY2* in VIGS-treated fruit using RT-PCR

Mature fruit were harvested from MY plants inoculated with pTRV2-GFP or pTRV2-PSY2 at 4 months after inoculation. Segments of the mature pericarp that displayed lighter colors were excised and their RNA was extracted. RT-PCR was conducted to verify the gene silencing using the following reaction mixture: 2 μl 4× diluted cDNA, 0.3 μl EX Taq (Takara), 2.5 μl 10× PCR buffer, 2 μl 10 mM dNTPs, 0.5 μl 10 pmol primers, and 17.2 μl triple-distilled water. The same primers used to amplify the entire *PSY2* gene were used (Supplementary Table S1). The RT-PCR conditions were 28 cycles of 95 °C for 30 s, 58 °C for 30 s, and 72 °C for 90 s. Three biological replicates were conducted, and *Actin* was used as the internal control.

### Carotenoid extraction and saponification of VIGS-treated fruit

Carotenoids were extracted as described by Yoo *et al.* (2017), with some modifications and under dim light. The samples were put on ice to prevent carotenoid oxidation and degradation. The lighter-colored pTRV2-PSY2-inoculated pericarps and the pTRV2-GFP-inoculated pericarps were diced and freeze-dried. Pieces from two TRV2-PSY2-inoculated fruits were pooled together to obtain enough tissue for extraction.

The freeze-dried tissues were ground, and ~50 mg of tissue powder was placed in a 2-ml tube with two glass beads. For the saponification, 600 μl tetrahydrofuran (THF), 375 μl petroleum ether, and 150 μl 25% NaCl was added to the samples, which were then vortexed and centrifuged for 3 min at 1500 *g* at 4 °C. The upper phase was transferred into a new 2-ml tube. Aliquots of 500 μl of petroleum ether were added to the remaining samples, which were then centrifuged, and the resulting second upper phase was combined with the first upper phase. The samples were then concentrated using a vacuum concentrator. The dried samples were dissolved with 500 μl HPLC-grade acetone (Honeywell) using sonication, filtered using a 0.2-μm syringe filter (Acrodisc LC 13 mm syringe filter, PVDF membrane; Pall, NY, USA), and bottled in a 1.5-mL HPLC amber vial. HPLC analysis of the carotenoids was performed by the NICEM chromatography laboratory (Seoul National University). Capsanthin, capsorubin, lutein, zeaxanthin, *α*-carotene, *β*-carotene, and *β*-cryptoxanthin were used as standards.

## Results

### MY harbors structural variations in *PSY1* and *CCS*

**Fig. 2.**
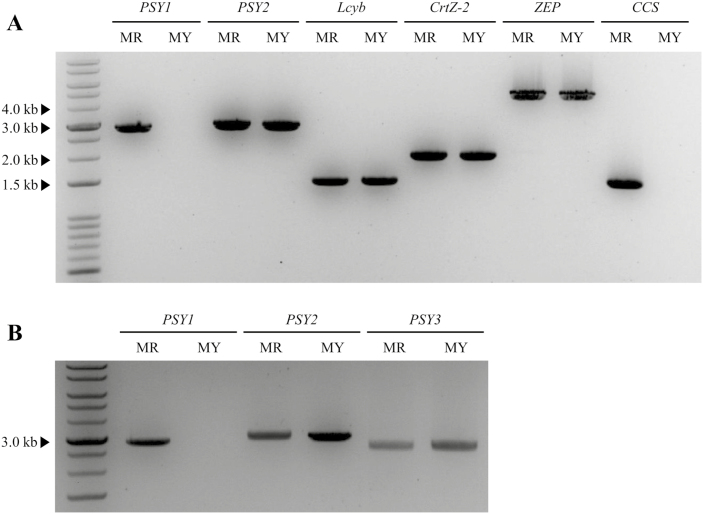
Polymorphism survey of carotenoid biosynthetic genes in the pepper genotypes ‘MicroPep Red’ (MR) and ‘MicroPep Yellow’ (MY). (A) gDNA amplification of six carotenoid biosynthetic genes using PCR. Primers were design to amplify the full length of each gene. The expected amplicon sizes were obtained for *PSY2*, *Lcyb*, *CrtZ-2*, and *ZEP*. No amplicon was obtained for *PSY1* and *CCS* in MY. (B) gDNA PCR amplification of the *PSY* genes. No amplicon was obtained for *PSY1* in MY. *PSY*, *phytoene synthase*; *Lcyb*, *lycopene ß-cyclase*; *CrtZ-2*, *ß-carotene hydroxylase*; *ZEP*, *zeaxanthin epoxidase*; *CCS*, *capsanthin-capsorubin synthase*.

Two *C. annuum* accessions, MR and MY, were used to identify the genetic factors that control the formation of yellow coloration in mature pepper fruit. The seven carotenoid biosynthetic genes *PSY1*, *PSY2*, *PSY3*, *Lcyb*, *CrtZ-2*, *ZEP* (*zeaxanthin epoxidase*), and *CCS*, were amplified from gDNA using PCR in order to identify differences between the two genotypes. The expected sizes of the amplicons are shown in Supplementary Table S1. In both MR and MY, the expected amplicon sizes were obtained for *PSY2*, *PSY3*, *Lcyb*, *CrtZ-2*, and *ZEP*, and no sequence variations were found in these genes between the accessions. In contrast, no amplicons were obtained from MY for *PSY1* and *CCS* (Fig. 2). These results indicated the possible existence of structural variations in *PSY1* and *CCS* in MY.

### MY has a complete deletion of *PSY1* and a transposon insertion in *CCS*

**Fig. 3.**
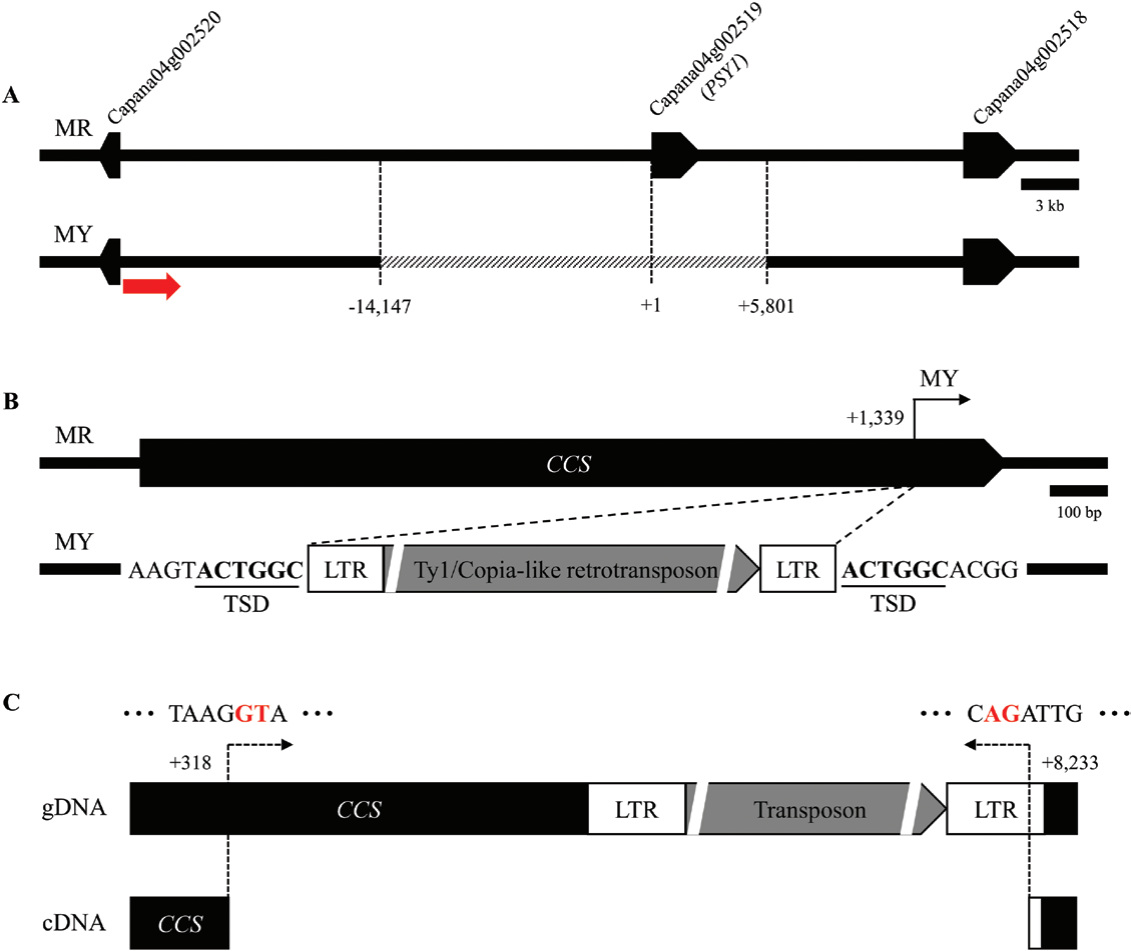
Structural variations of *PSY1* and *CCS* in pepper ‘MicroPep Yellow’ (MY) and ‘MicroPep Red’ (MR). (A) Genome-walking from *Capana04g002520* identifies a deletion of almost 20 kb spanning the *PSY1* genomic region. The primer used for the first genome-walking run is indicated by the arrow. (B) A Ty1/Copia-like retrotransposon insertion of almost 7 kb was discovered 1339 nt along *CCS* in MY. LTR, long terminal repeat; TSD, target site duplication. (C) The transcriptional variant of *CCS* in MY. Compared with the full *CCS* sequence in MR, much smaller bands were amplified from MY using RT-PCR. This transcriptional variation was regarded as being caused by the provision of a conserved sequence in the splicing junction by the inserted sequence. The letters underlined represent the conserved sequences of the exon–intron junction. The dotted lines and arrows indicate the genomic region that is spliced out during transcription.

To identify the possible structural variation of *PSY1* in MY, various regions of the gene, including the upstream and downstream sequences, were amplified using 12 sets of primers. No fragments of the expected size were obtained indicating the existence of a large insertion or a deletion in the gene. We then performed genome-walking to examine this. Since target-specific primers are required for genome-walking, the first primer set was designed using the gene *Capana04g0025200*, which is adjacent to *PSY1*. The ‘Zunla’ reference was used in the genome-walking, as the sequenced intergenic regions of *PSY1* were found there, whereas they were not present in the ‘CM334’ reference genome (Sol Genomics Network). Using several rounds of genome-walking, a 19 948-bp deletion including the entire *PSY1* genomic region was identified in the MY genome (Fig. 3A). To test the commonality of this mutation among *C. annuum* germplasm, we genotyped the 18 accessions that show yellow fruit color (Jeong *et al.*, 2019) using SCAR markers (Supplementary Table S2) and found that 15 of them had the same structural mutation as MY (Supplementary Fig. S5).

To investigate the structural variation of *CCS* in MY, long-range PCR was conducted. Several regions of *CCS* were amplified normally indicating the presence of a large insertion that could not be detected using regular PCR conditions. To overcome this limitation, the extension time of the PCR cycle was increased to 10 min, resulting in the amplification of a sequence containing a 6750-bp insertion located 1339 nt along *CCS* in MY (Fig. 3B). When this amplicon was sequenced and analysed, the inserted sequence was observed to contain a Ty1/Copia-like retrotransposon, ~1-kb long-terminal repeat (LTR) sequences, and a 6-bp target site duplication sequence (TSD)(ACTGGC) (Supplementary Dataset S1).

### MY produces a transcriptional variant of *CCS*

*CCS* consists of one exon with no intron. As MY contained the full *CCS* sequence, although interrupted by the transposon, RT-PCR was performed to examine whether functional transcripts of *CCS* were present in these plants. In MR, a 1497-bp amplicon was obtained. By contrast, a much smaller amplicon (513 bp) was obtained from MY. A sequence analysis of this transcript showed that most of the transposon, along with 1 kb of *CCS*, were spliced out in MY (Fig. 3C). Such transcriptional variation might result from the provision of a conserved sequence in the splicing junction (5′-GT/AG-3′) by the inserted transposon sequence (Brown, 1986), which might be functional as an active splicing junction of *CCS*. The resulting transcripts would be expected to encode a truncated protein containing 115 amino acids, whereas functional CCS comprises 498 amino acids.

### Mutations in *PSY1* and *CCS* control fruit color

To examine whether the structural variations of *PSY1* and *CCS* could result in the yellow fruit color of MY, an inheritance study was performed using a segregating population derived from a cross between MR and MY. All fruit from the F_1_ progeny were red, indicating that this color is dominant over yellow. In the F_2_ population, red, orange, and yellow fruits showed a segregation ratio of 156:111:14 (Table 1). This was consistent with an expected ratio of 9:6:1 (*χ*^2^=1.26, *P*=0.59), suggesting that the two genes interacted epistatically to determine the fruit color in this population.

**Table 1.** Segregation of phenotypes in the MR × MY F_2_ population

Population	Total plants	Mature fruit color			Expected ratio	*χ* ^2^
		Red	Orange	Yellow		
MR × MY F_2_	281	156	111	14	9:6:1	1.26

To examine whether *PSY1* and *CCS* were involved in the determination of fruit color, SCAR markers were used to genotype the F_2_ individuals (Supplementary Fig. S3). Plants containing the wild-type alleles (*PSY1*/*–* and *CCS*/*–*) of both genes produced red fruit. By contrast, yellow fruit were produced by plants containing homozygous recessive alleles for both genes (*psy1*/*psy1 ccs*/*ccs*). In plants containing either the *PSY1/– ccs/ccs* or *psy1/psy1 CCS/–* genotypes, the fruit were orange in color (Table 2).

**Table 2.** Co-segregation of genotypes and phenotypes in the MR × MY F_2_ population

Population	Size	Red, *PSY1/– CCS/–*	Orange, *PSY1/– ccs/ccs*	Orange, *psy1/psy1 CCS/–*	Yellow, *psy1/psy1 ccs/ccs*	Expected ratio	*χ* ^2^
MR × MY F_2_	281	156	48	63	14	9:3:3:1	3.27

### Orange pepper fruit have mutations in either *PSY1* or *CCS*

**Fig. 4.**
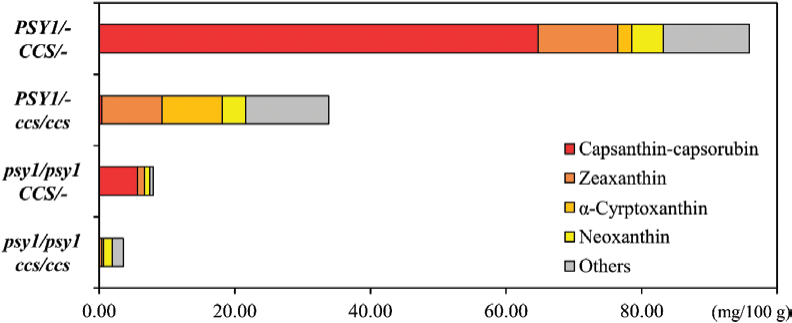
Carotenoid profiles of each of the F_2_ genotypes of the cross between pepper ‘MicroPep Yellow’ × ‘MicroPep Red’. The F_2_ individuals were genotyped by combination of *PSY1* and *CCS* alleles. Among the 11 carotenoid components examined, only the major ones are presented individually and the rest are grouped together as ‘Others’. Data are means of three biological replicates. SCAR markers were used to genotype the F_2_ individuals (Supplementary Fig. S3).

No phenotypic differences were observed between the orange-colored fruits produced by plants with the different genotypes *PSY1/– ccs/ccs* or *psy1*/*psy1 CCS/–*. For each genotypic group, UPLC was performed to determine the carotenoid profiles. The major carotenoid component of plants with the *PSY1*/*– CCS*/*–* genotype was capsanthin, whereas neoxanthin was the main component in the plants harboring *psy1/psy1 ccs/ccs* were (Fig. 4). Two distinct differences were identified between the orange-colored fruit produced by plants containing the different genotypes. First, the total carotenoid content of the fruit of the *PSY1/– ccs/ccs* genotype was four times higher than that of the fruit of the *psy1*/*psy1 CCS/–* genotype. Second, both capsanthin and capsorubin, the typical red pigments present in red peppers, were almost undetectable in the fruit of the *PSY1/– ccs/ccs* genotype (0.35 mg per 100 g DW), whereas these pigments accounted for almost 70% of the total carotenoids in the *psy1/psy1 CCS/–* fruit (5.67 mg per 100 g DW). These results indicated that, although the visible colors were similar between the two orange genotypes, the composition of their pigments were different.

In the *psy1* knockout plants (*psy1*/*psy1*), the carotenoids accumulated in the fruit regardless of the *CCS* genotype, although the total carotenoid content was ~10-fold lower than that of the plants containing a functional *PSY1* gene. In contrast to the *PSY1/– ccs/ccs* plants, the content of each carotenoid component was decreased in the *psy1/psy1 ccs/ccs* genotype. A similar trend was also observed in the *psy1/psy1 CCS/–* plants compared with the *PSY1/– CCS/–* plants, and the levels of most carotenoids except antheraxanthin were decreased. Thus, despite the impaired activity of PSY1, the carotenoid composition of the *psy1/psy1* plants was similar to the wild-type, although the individual levels were reduced (Fig. 4). The absolute carotenoid levels are shown in Supplementary Table S3. The results implied that other genes, such as the *PSY* homologs, might supplement the function of *PSY1*, regardless of *CCS*.

### *PSY2* is expressed in pepper fruit

**Fig. 5.**
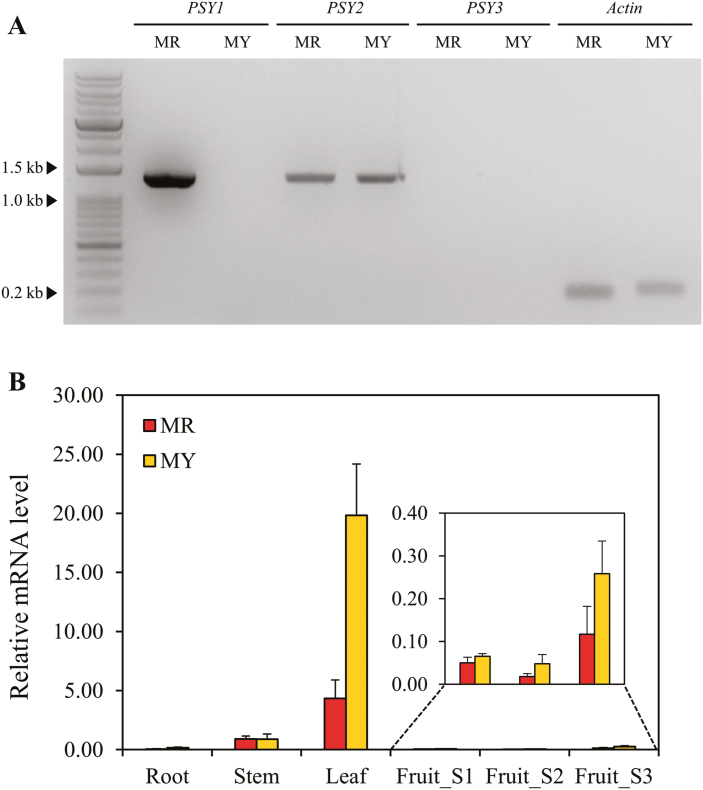
Expression pattern of the *PSY* genes in various tissues of pepper ‘MicroPep Yellow’ (MY) and ‘MicroPep Red’ (MR). (A) Expression analyses of the three *PSY* genes in mature fruit pericarps. *Actin* served as the control. (B) Expression analysis of *PSY2* in various tissues. Relative transcript levels of *PSY2* were determined using qRT-PCR, and normalized to the expression of *Actin*. The data are the means (±SD) of three independent experiments. The fruit developmental stages were: S1, immature stage; S2, breaker stage; and S3, mature stage.

Based on the UPLC data, we postulated that *PSY2* or *PSY3* might be involved in carotenoid biosynthesis in the pepper fruit. To test this hypothesis, we analysed the expression levels of the *PSY* homologs in mature fruit pericarps using RT-PCR, and found that *PSY1* transcripts could only be detected in MR (Fig. 5A). *PSY2* transcripts of the expected size were obtained from both MR and MY, while *PSY3* transcripts were not detected in either accession. These results demonstrated that *PSY2* was expressed in the fruit and that may be involved in color formation. A further analysis using qRT-PCR confirmed that *PSY2* was expressed in the pericarp tissues, although the highest levels of expression were detected in the leaves (Fig. 5B). The *PSY2* expression levels in the leaves and fruits were significantly higher in MY than in MR, indicating that *PSY2* may compensate for the loss of *PSY1* function in MY.

### PSY2 has the same enzymatic function as PSY1

**Fig. 6.**
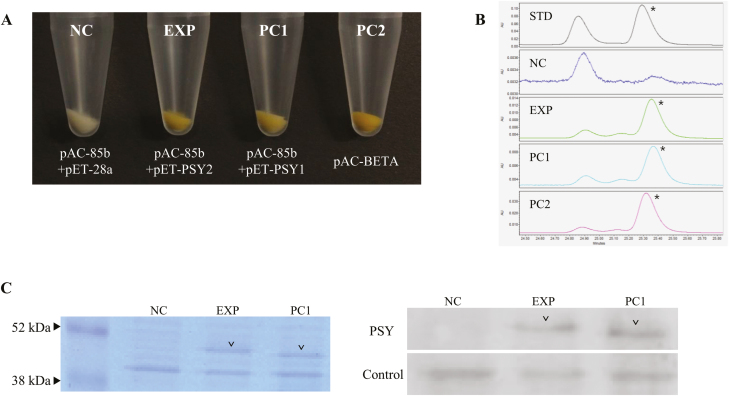
Complementation assay for pepper *PSY2* in *E. coli*. (A) Expression of *PSY2* or *PSY1* in *E. coli* strains carrying pAC-85b. An *E. coli* strain carrying the empty vector alone served as the negative control (NC). The same *E. coli* strain containing pAC-85b and pET-PSY1 was used as a positive control (PC1), and a *β*-carotene-accumulating *E. coli* strain (pAC-BETA) served as a positive control for the color formation (PC2). The same *E. coli* strain with pAC-85b and pET-PSY2 is indicated as EXP (experimental group). The cell was harvested by centrifugation to transparent 1.5 mL tubes to show the colors. (B) UPLC analysis of carotenoids extracted from each of the samples shown in (A). The *β*-carotene peaks are indicated with asterisks. AU, arbitrary absorbance units; STD, standard mixture of *α*- and *β*-carotenes. (C) Western blot analysis of *PSY* expression in transformed *E. coli*. Coomassie-stained SDS-PAGE gel (left) and Western blot (right) of the NC, EXP, and the PC1 samples using anti-PSY antibodies. The EXP and PC1-specific bands are indicated by arrowheads.

The enzymatic function of PSY2 was examined using a complementation assay in *E. coli* BL21(DE3) cells carrying pAC-85b and pET-PSY2 (EXP). The same *E. coli* strain containing pAC-85b and pET-PSY1 was used as a positive control (PC1), while cells containing pAC-85b and the empty pET-28a(+) vector were used as the negative control (NC). For comparison of the colored pigments produced by these cells, transformants producing *β*-carotene (pAC-BETA) were also used (Supplementary Fig. S2). If PSY2 is able to biosynthesize the carotenoid backbone, the *E. coli* pellets would be orange in color due to the resulting *β*-carotene content. As shown in Fig. 6A, the *E. coli* pellets from EXP were orange, as were those of the two positive control groups, PC1 and PC2. UPLC analysis of the *E. coli* extracts was conducted to confirm that the orange color of the experimental cells expressing *PSY2* was due to the accumulation of *β*-carotene. A clear *β*-carotene peak was detected in all cells except the negative control group (Fig. 6B). The *β*-carotene contents were 1.57 mg per 100 g and 2.23 mg per 100 g FW in the cells containing pET-PSY2 and pET-PSY1, respectively. These results demonstrated that PSY2 has a similar catalytic activity to PSY1 in carotenoid biosynthesis.

To confirm the production of the PSY proteins in the *E. coli* cells, an immunoblot assay was conducted. A 2-ml aliquot of each cell culture was sampled, treated with SDS sample buffer, and run on a 12% SDS-PAGE gel. One of the two replicated gels were stained with Coomassie Blue. The specific band sizes of EXP with pET-PSY2 and PC1 with pET-PSY1 were between 38–52 kDa (Fig. 6C). The other replicated sample was transferred onto a nitrocellulose membrane and treated with an antibody against tomato SlPSY1, which showed a high amino acid sequence similarity with CaPSY1 (90.66%) and CaPSY2 (82.96%) (Supplementary Fig. S4). Specific bands of ~48 kDa were detected in both EXP and PC1. The EXP band was slightly bigger than that of PC1 (48.44 kDa and 47.07 kDa, respectively), consistent with the expected sizes of CaPSY2 and CaPSY1 (Fig. 6C). These results demonstrated that the recombinant proteins were expressed in *E. coli*.

### Silencing of *PSY2* leads to lighter yellow fruit color

**Fig. 7.**
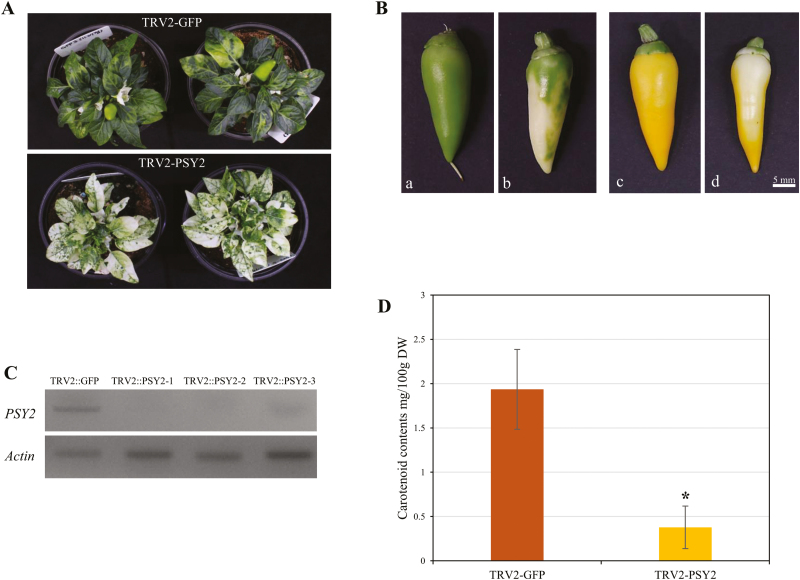
Virus-induced gene silencing (VIGS) of *PSY2* in pepper ‘MicroPep Yellow’ (MY). (A) Phenotypes of plants inoculated with TRV2-GFP (vector control; top) and TRV2-PSY2 (bottom) at 60 d after inoculation. (B) Effect of VIGS of *PSY2* on the color of (a, b) immature and (c, d) mature fruit that were inoculated with (a, c) TRV2-GFP or (b, d) TRV2-PSY2. (C) Expression analysis of *PSY2* using RT-PCR. Three biological replicates of *PSY2*-silenced fruits were used (1–3). *Actin* served as the control. (D) Total carotenoid contents of fruit inoculated with TRV2-GFP or TRV2-PSY2. The significant difference was determined using a Student’s *t*-test: *P<0.01.

The function of *PSY2* was evaluated in MY plants using TRV-mediated VIGS, with *Agrobacterium* cells carrying pTRV2-GFP being used as a negative control. The symptoms of *PSY2* silencing were visualized at 15 d after inoculation (DAI). The plants inoculated with pTRV2-GFP did not display any significant differences to the wild-type. The *PSY2*-silenced plants had a mosaic phenotype of pale and green patches on the leaves, due to the characteristics of the TRV vector (Fig. 7A). The flowers and fruits developed at about 60 DAI and 75 DAI, respectively. There were no visible changes in the flower colors of both groups, but a slightly lighter peduncle color was observed in the plants inoculated with TRV2-PSY2. In the plants inoculated with pTRV2-GFP, the immature fruit were a dark green color, whereas those of the *PSY2-*silenced plants were white to pale green (Fig. 7B). The mature fruit color also showed a similar trend, as the fruits inoculated with pTRV2-GFP showed an intense yellow color, whereas the *PSY2-*silenced fruits were white to ivory.

RT-PCR was conducted to compare the expression levels of *PSY2* in the mature fruit of the pTRV2-GFP-inoculated and *PSY2*-silenced plants. While the fruits of the plants inoculated with pTRV2-GFP expressed *PSY2*, there was almost no detectable expression in the *PSY2-*silenced fruit tissues (Fig. 7C), indicating successful post-transcriptional gene-silencing (PTGS) of the target gene.

HPLC analysis was conducted to obtain quantitative and qualitative analyses of the carotenoid contents in the mature fruits inoculated with TRV-GFP and TRV2-PSY2. The total carotenoid contents of TRV2-GFP and TRV2-PSY2 were 1.94 mg per 100g DW and 0.38 mg per 100g DW, respectively (Fig. 7D). The mature fruits of the control plants had a greater than 5-fold higher carotenoid content than the *PSY2*-silenced fruits. Lutein was found to be the most prominent component in both TRV2-GFP and TRV2-PSY2 (Supplementary Fig. S6).

## Discussion

In this study, we determined that the MY pepper line, which produces yellow mature fruit, has structural mutations in *PSY1* and *CCS*. Yellow fruit color that results from mutations in the *CCS* coding region in pepper has previously been reported in a number of studies (Lefebvre *et al.*, 1998; Popovsky and Paran 2000; Li *et al.*, 2013). Our study also indicated the presence of a mutation in the *CCS* coding region, which resulted in transcriptional variant of 115 amino acids (Fig. 3). Splicing variations and mutations in the coding sequence of *PSY1* have been examined previously (Kim *et al.*, 2010; Jeong *et al.*, 2019); however, we provide the first report of the complete deletion of *PSY1* (Fig. 2). If PSY1 were the only enzyme able to catalyse the first step of carotenoid biosynthesis, the *psy1* knockout plants would not be able to produce any; however, we detected low levels of carotenoids in the knockout plants. *PSY2* was expressed in the pericarp tissues of both the wild-type and *psy1* knockout plants, and we verified the enzymatic activity of PSY2 using a color complementation assay (Fig. 6). A VIGS analysis also showed that knockdown of *PSY2* resulted in a lighter yellow color of mature fruit of *psy1* mutant pepper (Fig. 7). Thus, we have demonstrated that *PSY2* controls the yellow fruit color in pepper *psy1* knockout mutants.

Tomato has three *phytoene synthase* genes, namely *SlPSY1*, *SlPSY2*, and *SlPSY3* (Fantini *et al.*, 2013). In contrast to *SlPSY1*, which is predominantly expressed in the fruit, most expression of *SlPSY2* is in the petals, followed by the leaves, sepals, and ovary tissues, and it is also expressed at a lower level in other tissues, including the roots and fruit (Giorio *et al.*, 2008). *SlPSY3* is expressed mainly in root tissues under stress conditions (Kachanovsky *et al.*, 2012). The silencing of *SlPSY2* and *SlPSY3* does not result in dramatic differences in the color of mature tomato fruit (Fantini *et al.*, 2013), suggesting that these genes have a negligible role in the pericarp tissues. However, the silencing of *PSY2* in tomato was conducted in a functional *PSY1* background, and hence the contribution of *PSY2* might have been masked by the activity of PSY1. In addition to *CaPSY1*, the pepper genome contains two more putative *PSY* genes, whose functions have not yet been identified. We hypothesized that *CaPSY2* might be involved in color formation in MY as *CaPSY3* was not amplified in MY fruit (Fig. 5A). Consistent with the expression pattern of *SlPSY2*, only a basal level of *CaPSY2* transcripts was observed in the fruit, regardless of the developmental stage examined (Fig. 5B). Meanwhile, the level of *CaPSY2* transcripts was much higher in MY in most tissues compared with the red-colored MR pepper line, indicating positive feedback and corresponding up-regulation of this gene in MY due to the absence of *CaPSY1*. We examined the enzymatic activity of CaPSY2 using a color complementation assay in *E. coli* (Fig. 6). Similar to previous studies that have used pAC-85b to confirm the activity of *PSY* homologs in other crops, we first investigated the color change of the cell pellet and then performed UPLC analysis of the cell extract. Both results demonstrated that CaPSY2 functions as an enzyme in carotenoid biosynthesis, and we conclude that CaPSY2 can supplement the activity of CaPSY1 and contribute to the development of the yellow color in the fruit of MY.

Using single-molecule real-time (SMRT) sequencing, Jeong *et al.* (2019) identified the sequence variations of six carotenoid biosynthetic genes, including *PSY1* and *PSY2*, in diverse *Capsicum* genotypes. Of the 94 *C. annuum* accessions they analysed, *PSY1* was not amplified in 18 lines. We hypothesized that these genotypes could have a large deletion of *PSY1*, as found in our present study. The same deletion of *PSY1* was found in 15 out of the 18 accessions, for which we had already observed no amplification of *PSY1* using SCAR markers designed in this study (Supplementary Fig. S5). The marker that we have developed in this study is therefore widely applicable for use in other *C. annuum* germplasms. In addition, Jeong *et al.* (2019) reported another three *psy1* knockout mutations in eight accessions, two of which are frame-shift mutations (*psy1-c5* and *psy1-c10*) and another is a nonsense mutation (*psy1-c9*). However, they identified no critical mutations in *PSY2*. These results also support our hypothesis that *PSY2* may complement the function of *PSY1* in accessions with non-functional *PSY1*. Further experiments will be required to obtain clear evidence of the exact function of *PSY2* in fruit color formation.

According to the three-locus model, the diverse colors of pepper fruit are determined by *PSY1*, *CCS*, and one unidentified locus (Lefebvre *et al.*, 1998; Huh *et al.*, 2001). It is not possible to state that *PSY2* is encoded this locus based solely on the results of our study, as we have not found a natural *PSY2* mutation in plants that already have mutant *PSY1*. Other aspects of carotenoid metabolism have recently been investigated for their role in fruit coloration in pepper, and several transcription factors have been shown to play a role in the color intensity of mature fruit. The Golden2-like transcription factor (GLK2) and Arabidopsis pseudo-response regulator 2-like (APRR2) affect plastid levels and thus regulate color in a quantitative manner in pepper fruit (Brand *et al.*, 2012; Pan *et al.*, 2013). For this reason, we used VIGS to silence *PSY2* expression in *psy1* knockout plants and this resulted in a white mosaic phenotype in both the leaves and fruit (Fig. 7), which supported the other results obtained in this study.

Using UPLC analysis, we examined the carotenoid profiles of two pepper genotypes that produce orange fruit (Fig. 4, Supplementary Table S3). The orange color of the *PSY1/– ccs/ccs* fruits resulted from moderate accumulation of diverse carotenoid components except for capsanthin and capsorubin, whereas the orange color of *psy1/psy1 CCS/–* fruits was generated by a proportionally high level of red pigments (52.0% for capsanthin, 18.9% for capsorubin), although their absolute contents were considerably lower than those of the *PSY1/– CCS/–* type. In the case of *PSY1/– CCS/–* red fruits, the proportion of these red pigments accounted for 67.5% of all carotenoids, which was slightly less than that of the pigments in the *psy1/psy1 CCS/–* orange fruits. This implies that it is not only the relative proportions of red and other carotenoid pigments that is important for the development of red fruit, but also the absolute content of the pigments.

In conclusion, in this study we have demonstrated that a candidate-gene approach combined with chemotyping via UPLC can be a useful tool for the identification of the genetic factors that control the carotenoid metabolic pathway. While the contribution of PSY2 seemed to be minimal in pepper fruit compared with that of functional PSY1, it appeared to play an essential role in *psy1* knockout plants, as it was able to allow a basal level of carotenoid accumulation. *PSY2* must therefore be considered alongside *PSY1* and *CCS* as an essential gene for the successful breeding of peppers with new color and carotenoid profiles.

## Supplementary data

Supplementary data are available at *JXB* online.

Table S1. List of primers used to amplify the carotenoid biosynthetic genes.

Table S2. List of primers used in the *PSY1* and *CCS* SCAR marker analyses.

Table S3. Absolute contents of carotenoids in the MR × MY F_2_ population.

Fig. S1. The carotenoid biosynthetic pathway in *Capsicum* spp.

Fig. S2. Details of vectors used in the color complementation assay.

Fig. S3. SCAR marker genotyping of *PSY1* and *CCS*.

Fig. S4. Amino acid sequence alignment of pepper and tomato PSY1 and PSY2.

Fig. S5. SCAR marker test of accessions from which *PSY1* could not be amplified.

Fig. S6. HPLC profiles of the carotenoid extracts obtained from the *PSY2* VIGS-treated MY plants.

Dataset S1. LTR retrotransposon sequences discovered in *CCS* of MY.

eraa155_suppl_Supplementary_Material

## References

[CIT0001] Al-Babili S, Bouwmeester HJ 2015 Strigolactones, a novel carotenoid-derived plant hormone. Annual Review of Plant Biology 66, 161–186. 10.1146/annurev-arplant-043014-114759 25621512

[CIT0002] Blas AL, Ming R, Liu Z, Veatch OJ, Paull RE, Moore PH, Yu Q 2010 Cloning of the papaya chromoplast-specific lycopene beta-cyclase, *CpCYC-b*, controlling fruit flesh color reveals conserved microsynteny and a recombination hot spot. Plant Physiology 152, 2013–2022. 20181753 10.1104/pp.109.152298 PMC2850019

[CIT0003] Borovsky Y, Tadmor Y, Bar E, Meir A, Lewinsohn E, Paran I 2013 Induced mutation in β*-CAROTENE HYDROXYLASE* results in accumulation of β-carotene and conversion of red to orange color in pepper fruit. Theoretical and Applied Genetics 126, 557–565. 23124390 10.1007/s00122-012-2001-9

[CIT0004] Bouvier F, Hugueney P, d’Harlingue A, Kuntz M, Camara B 1994 Xanthophyll biosynthesis in chromoplasts: isolation and molecular cloning of an enzyme catalyzing the conversion of 5,6-epoxycarotenoid into ketocarotenoid. The Plant Journal 6, 45–54. 7920703 10.1046/j.1365-313x.1994.6010045.x

[CIT0005] Brand A, Borovsky Y, Meir S, Rogachev I, Aharoni A, Paran I 2012 *pc8.1*, a major QTL for pigment content in pepper fruit, is associated with variation in plastid compartment size. Planta 235, 579–588. 21987007 10.1007/s00425-011-1530-9

[CIT0006] Brown JW 1986 A catalogue of splice junction and putative branch point sequences from plant introns. Nucleic Acids Research 14, 9549–9559. 3808952 10.1093/nar/14.24.9549 PMC341320

[CIT0007] Cunningham FX Jr, Gantt E 2007 A portfolio of plasmids for identification and analysis of carotenoid pathway enzymes: *Adonis aestivalis* as a case study. Photosynthesis Research 92, 245–259. 17634749 10.1007/s11120-007-9210-0

[CIT0008] Cunningham FX Jr, Pogson B, Sun Z, McDonald KA, DellaPenna D, Gantt E 1996 Functional analysis of the beta and epsilon lycopene cyclase enzymes of *Arabidopsis* reveals a mechanism for control of cyclic carotenoid formation. The Plant Cell 8, 1613–1626. 8837512 10.1105/tpc.8.9.1613 PMC161302

[CIT0009] Domonkos I, Kis M, Gombos Z, Ughy B 2013 Carotenoids, versatile components of oxygenic photosynthesis. Progress in Lipid Research 52, 539–561. 23896007 10.1016/j.plipres.2013.07.001

[CIT0010] Fantini E, Falcone G, Frusciante S, Giliberto L, Giuliano G 2013 Dissection of tomato lycopene biosynthesis through virus-induced gene silencing. Plant Physiology 163, 986–998. 24014574 10.1104/pp.113.224733 PMC3793073

[CIT0011] Fraser PD, Bramley PM 2004 The biosynthesis and nutritional uses of carotenoids. Progress in Lipid Research 43, 228–265. 15003396 10.1016/j.plipres.2003.10.002

[CIT0012] Fu X, Feng C, Wang C, Yin X, Lu P, Grierson D, Xu C, Chen K 2014 Involvement of multiple phytoene synthase genes in tissue- and cultivar-specific accumulation of carotenoids in loquat. Journal of Experimental Botany 65, 4679–4689. 24935622 10.1093/jxb/eru257 PMC4115255

[CIT0013] Giorio G, Stigliani AL, D’Ambrosio C 2008 Phytoene synthase genes in tomato (*Solanumlycopersicum* L.) – new data on the structures, the deduced amino acid sequences and the expression patterns. The FEBS Journal 275, 527–535. 18167141 10.1111/j.1742-4658.2007.06219.x

[CIT0014] Guzman I, Hamby S, Romero J, Bosland PW, O’Connell MA 2010 Variability of carotenoid biosynthesis in orange colored *Capsicum* spp. Plant Science 179, 49–59. 20582146 10.1016/j.plantsci.2010.04.014 PMC2889374

[CIT0015] Ha SH, Kim JB, Park JS, Lee SW, Cho KJ 2007 A comparison of the carotenoid accumulation in *Capsicum* varieties that show different ripening colours: deletion of the *capsanthin-capsorubin synthase* gene is not a prerequisite for the formation of a yellow pepper. Journal of Experimental Botany 58, 3135–3144. 17728301 10.1093/jxb/erm132

[CIT0016] Huh JH, Kang BC, Nahm SH, Kim S, Ha KS, Lee MH, Kim BD 2001 A candidate gene approach identified *phytoene synthase* as the locus for mature fruit color in red pepper (*Capsicum* spp.). Theoretical and Applied Genetics 102, 524–530.

[CIT0017] Hurtado-Hernandez H, Smith PG 1985 Inheritance of mature fruit color in *Capsicum annuum* L. Journal of Heredity 76, 211–213.

[CIT0018] Jackson H, Braun CL, Ernst H 2008 The chemistry of novel xanthophyll carotenoids. The American Journal of Cardiology 101, 50D–57D. 10.1016/j.amjcard.2008.02.008 18474275

[CIT0019] Jeong HB, Kang MY, Jung A, Han K, Lee JH, Jo J, Lee HY, An JW, Kim S, Kang BC 2019 Single-molecule real-time sequencing reveals diverse allelic variations in carotenoid biosynthetic genes in pepper (*Capsicum* spp.). Plant Biotechnology Journal 17, 1081–1093. 30467964 10.1111/pbi.13039 PMC6523600

[CIT0020] Kachanovsky DE, Filler S, Isaacson T, Hirschberg J 2012 Epistasis in tomato color mutations involves regulation of *phytoene synthase 1* expression by *cis*-carotenoids. Proceedings of the National Academy of Sciences, USA 109, 19021–19026. 10.1073/pnas.1214808109 PMC3503155 23112190

[CIT0021] Kang B, Gu Q, Tian P, Xiao L, Cao H, Yang W 2014 A chimeric transcript containing *Psy1* and a potential mRNA is associated with *yellow flesh* color in tomato accession PI 114490. Planta 240, 1011–1021. 24663441 10.1007/s00425-014-2052-z

[CIT0022] Kim J, Park M, Jeong ES, Lee JM, Choi D 2017 Harnessing anthocyanin-rich fruit: a visible reporter for tracing virus-induced gene silencing in pepper fruit. Plant Methods 13, 3. 28053648 10.1186/s13007-016-0151-5 PMC5209810

[CIT0023] Kim JS, An CG, Park JS, Lim YP, Kim S 2016 Carotenoid profiling from 27 types of paprika (*Capsicum annuum* L.) with different colors, shapes, and cultivation methods. Food Chemistry 201, 64–71. 26868549 10.1016/j.foodchem.2016.01.041

[CIT0024] Kim OR, Cho MC, Kim BD, Huh JH 2010 A splicing mutation in the gene encoding *phytoene synthase* causes orange coloration in Habanero pepper fruits. Molecules and Cells 30, 569–574. 21120629 10.1007/s10059-010-0154-4

[CIT0025] Kim YK, Kim S, Um JH, et al 2013 Functional implication of *β*-carotene hydroxylases in soybean nodulation. Plant Physiology 162, 1420–1433. 23700351 10.1104/pp.113.215020 PMC3707551

[CIT0026] Lefebvre V, Kuntz M, Camara B, Palloix A 1998 The *capsanthin-capsorubin synthase* gene: a candidate gene for the y locus controlling the red fruit colour in pepper. Plant Molecular Biology 36, 785–789. 9526511 10.1023/a:1005966313415

[CIT0027] Li Z, Wang S, Gui XL, Chang XB, Gong ZH 2013 A further analysis of the relationship between yellow ripe-fruit color and the *capsanthin-capsorubin synthase* gene in pepper (*Capsicum* sp.) indicated a new mutant variant in *C. annuum* and a tandem repeat structure in promoter region. PLoS ONE 8, e61996. 23637942 10.1371/journal.pone.0061996 PMC3630222

[CIT0028] López-Emparán A, Quezada-Martinez D, Zúñiga-Bustos M, Cifuentes V, Iñiguez-Luy F, Federico ML 2014 Functional analysis of the *Brassica napus* L. phytoene synthase (PSY) gene family. PLoS ONE 9, e114878. 25506829 10.1371/journal.pone.0114878 PMC4266642

[CIT0029] Nambara E, Marion-Poll A 2005 Abscisic acid biosynthesis and catabolism. Annual Review of Plant Biology 56, 165–185. 10.1146/annurev.arplant.56.032604.144046 15862093

[CIT0030] Pan Y, Bradley G, Pyke K, et al 2013 Network inference analysis identifies an *APRR2-Like* gene linked to pigment accumulation in tomato and pepper fruits. Plant Physiology 161, 1476–1485. 23292788 10.1104/pp.112.212654 PMC3585610

[CIT0031] Paran I, van der Knaap E 2007 Genetic and molecular regulation of fruit and plant domestication traits in tomato and pepper. Journal of Experimental Botany 58, 3841–3852. 18037678 10.1093/jxb/erm257

[CIT0032] Perry KL, Simonitch TA, Harrison-Lavoie KJ, Liu ST 1986 Cloning and regulation of *Erwinia herbicola* pigment genes. Journal of Bacteriology 168, 607–612. 3023282 10.1128/jb.168.2.607-612.1986 PMC213523

[CIT0033] Popovsky S, Paran I 2000 Molecular genetics of the *y* locus in pepper: its relation to *capsanthin-capsorubin synthase* and to fruit color. Theoretical and Applied Genetics 101, 86–89.

[CIT0034] Porebski S, Bailey LG, Baum BR 1997 Modification of a CTAB DNA extraction protocol for plants containing high polysaccharide and polyphenol components. Plant Molecular Biology Reporter 15, 8–15.

[CIT0035] Rodríguez-Suárez C, Atienza SG, Pistón F 2011 Allelic variation, alternative splicing and expression analysis of *Psy1* gene in *Hordeum chilense* Roem. et Schult. PLoS ONE 6, e19885. 21603624 10.1371/journal.pone.0019885 PMC3095628

[CIT0036] Sun T, Yuan H, Cao H, Yazdani M, Tadmor Y, Li L 2018 Carotenoid metabolism in plants: the role of plastids. Molecular Plant 11, 58–74. 28958604 10.1016/j.molp.2017.09.010

[CIT0037] Tian SL, Li L, Chai WG, Shah SN, Gong ZH 2014 Effects of silencing key genes in the capsanthin biosynthetic pathway on fruit color of detached pepper fruits. BMC Plant Biology 14, 314. 25403855 10.1186/s12870-014-0314-3 PMC4245796

[CIT0038] Wang H, Ou C, Zhuang F, Ma Z 2014 The dual role of phytoene synthase genes in carotenogenesis in carrot roots and leaves. Molecular Breeding 34, 2065–2079 26316840 10.1007/s11032-014-0163-7 PMC4544633

[CIT0039] Yoo H, Park W, Lee GM, Oh CS, Yeam I, Won DC, Kim CK, Lee J 2017 Inferring the genetic determinants of fruit colors in tomato by carotenoid profiling. Molecules 22, 764. 10.3390/molecules22050764 PMC6154295 28481314

[CIT0040] Yuan H, Zhang J, Nageswaran D, Li L 2015 Carotenoid metabolism and regulation in horticultural crops. Horticulture Research 2, 15036. 26504578 10.1038/hortres.2015.36 PMC4591682

