# Supplementary material for: Phytoene synthase 2 can compensate for the absence of PSY1 in the control of color in Capsicum fruit
Source: J Exp Bot. 2020 Mar 27;71(12):3417–27. doi: 10.1093/jxb/eraa155 (PMC7475241; doi:10.1093/jxb/eraa155)
Supplement: eraa155_suppl_Supplementary_Material [file eraa155_suppl_supplementary_material.pdf]

Supplementary table

Table S1. List of primers used in the SCAR marker analyses of *PSY1* and *CCS*.

Marker	Primer sequence (5′ to 3′)	Position (Zunla Ch4)	Amplicon (bp)
PSY1-MR-F	ATGGTTGTGCTGACAATGATTGATG	205,345,058-082	1,187
PSY1-MY-F	GATTTTGAATCTCCAACACACAAGC	205,325,421-445	876
PSY1-COM-R	GAGTCCACATTAGCAAAACACATGA	205,346,244-220	-
CCS-MR-F	AATGTTATGGCTATTGGTGGGACTT	227,702,924-900	374
CCS-MY-F	TGCAGTGAACTGCTGCAGAAAC	Not available	787
CCS-COM-R	TTAGAGGCATGTCCAAAAAGGTACA	227,702,551-575	-

Table S2. Absolute carotenoids content of MR x MY F2 population.

mg/100g	**MR**	**MY**	***PSY1/- CCS/-***	***PSY1/- ccs/ccs***	***psy1/psy1 CCS/-***	***psy1/psy1 ccs/ccs***
Neoxanthin	4.46±0.04	0.20±0.00	4.70±0.40	3.39±0.73	0.78±0.03	1.22±0.17
Capsorubin	15.16±1.54	0.01±0.00	19.48±2.24	0.00±0.00	1.51±0.21	0.00±0.00
Violaxanthin	0.00±0.00	0.00±0.00	2.64±0.31	0.64±0.01	0.00±0.00	0.39±0.01
Capsanthin	66.92±5.12	0.38±0.00	45.26±4.53	0.35±0.01	4.16±0.52	0.00±0.00
Antheraxanthin	0.00±0.00	0.16±0.00	0.00±0.00	0.56±0.00	0.24±0.01	0.17±0.00
Zeaxanthin	29.32±3.73	0.22±0.00	11.76±0.22	8.94±0.54	1.05±0.23	0.41±0.02
Lutein	0.00±0.00	0.32±0.01	0.34±0.02	3.18±0.03	0.27±0.01	0.55±0.01
α-cyrptoxanthin	0.67±0.13	0.13±0.05	2.01±0.28	8.89±0.77	0.00±0.00	0.27±0.01
β-cryptoxanthin	1.43±0.30	0.12±0.01	2.03±0.36	1.25±0.02	0.00±0.00	0.16±0.01
α-carotene	2.67±0.01	0.12±0.01	3.39±0.39	5.27±0.36	0.00±0.00	0.10±0.02
β-carotene	5.01±1.04	0.27±0.01	4.29±0.37	1.34±0.12	0.00±0.00	0.28±0.02
Total carotenoids	125.65±11.63	1.93±0.06	95.90±9.12	33.82±1.11	8.00±1.01	3.55±0.21

Supplementary figure legends

Fig. S1. Carotenoid biosynthetic pathway in *Capsicum* spp. IPP, isopentenyl diphosphate; DMAPP, dimethylallyl diphosphate; GGPP, geranylgeranyl diphosphate; PSY, phytoene synthase; PDS, phytoene desaturase; ZISO, ζ-carotene isomerase; CRISTO, carotenoid isomerase; LCYE, lysopene ε-cyclase; LCYB, lycopene β-cyclase; CrtZ-2, β-carotene hydroxylase; ZEP, zeaxanthin epoxidase; NXS, neoxanthin synthase; CCS, capsanthin-capsorubin synthase. Carotenoid names are colored according to its respective colors, while black means no color. Arrows indicate biosynthesis.

Fig. S2. Vector information used in the color complementation assay. pAC-ETA contains the *CrtE, CrtY, CrtI*, and *CrtB* carotenoid pathway genes from *Erwinia herbicola*, and thus causes β-carotene production in *E. coli*. The pAC-85b vector lacks *CrtB,* a *PSY* homolog, and cannot cause the biosynthesis of β-carotene. For the expression of *PSY2* derived from MicroPep, the pET-28a(+) vector containing the *T7* promoter was used.

Fig. S3. SCAR marker genotyping of *PSY1* and *CCS*. Marker genotype of *PSY1* (A) and *CCS* (B) in the parental lines MR and MY, an F_1 hybrid, and six representative F_2 plants. For *PSY1*, a bigger band (1,187 bp) is a representative of the MR-type allele, while a smaller band (374 bp) is a representative of the MY-type allele in *CCS*.

Fig. S4. Amino acid sequence alignment of pepper and tomato PSY1 and PSY2. Asterisks (*) indicate an identical residue between the sequences.

Fig. S5. SCAR marker genotyping of the accessions from which *PSY1* was not previously amplified in study of Jeong *et al.* (2018). (A) The primer binding sites in a genomic diagram. Common forward primer (blue) and distinct reverse primers (red and yellow, respectively) were used for each template. (B) Results of the SCAR marker test. Among the 18 accessions in which *PSY1* was not amplified, 15 were revealed to possess a structural variation identical to MY. The other three accessions were not amplified.

Fig. S6. HPLC profiles of the carotenoid extracts obtained from the VIGS-treated MY plants. Both TRV2-GFP- (left) and TRV2-PSY2-inoculated (right) mature fruits contained lutein as a major component. Au, absorbance units.

4

Supplementary figures

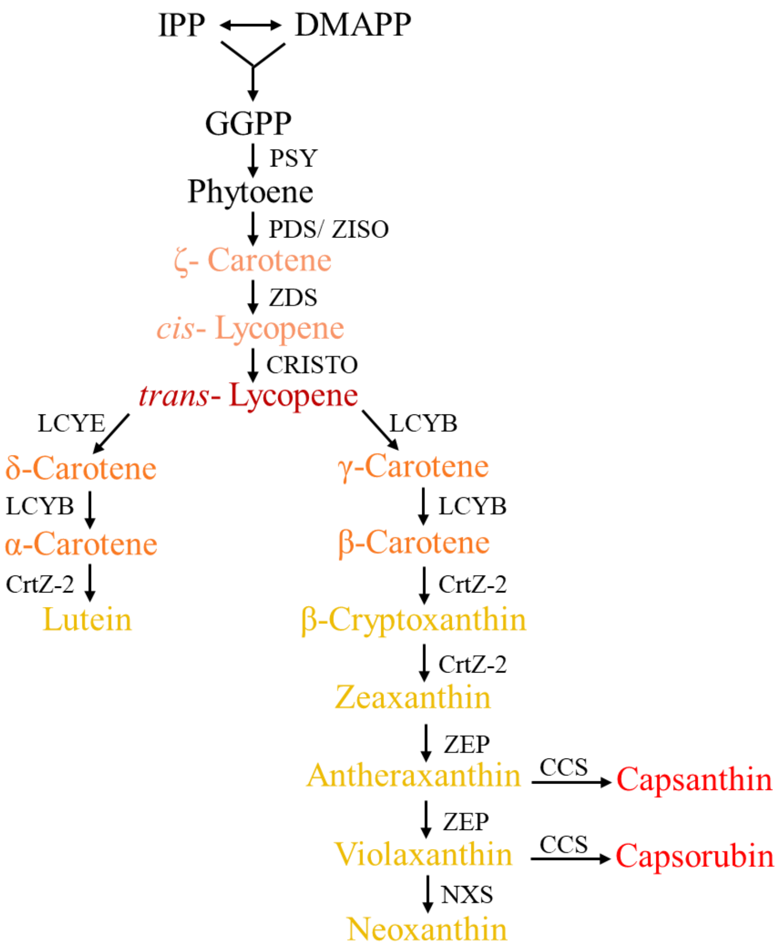

Fig. S1. Carotenoid biosynthetic pathway in *Capsicum* spp.

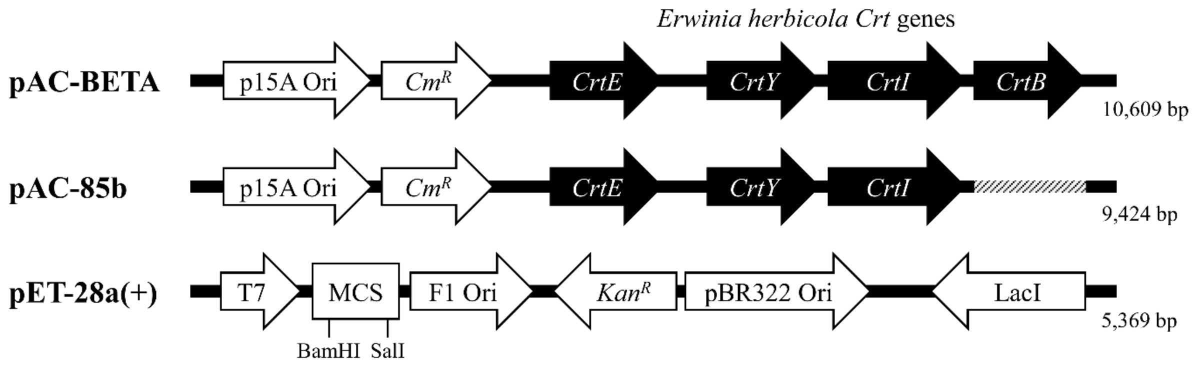

Fig. S2. Vector information used in the color complementation assay.

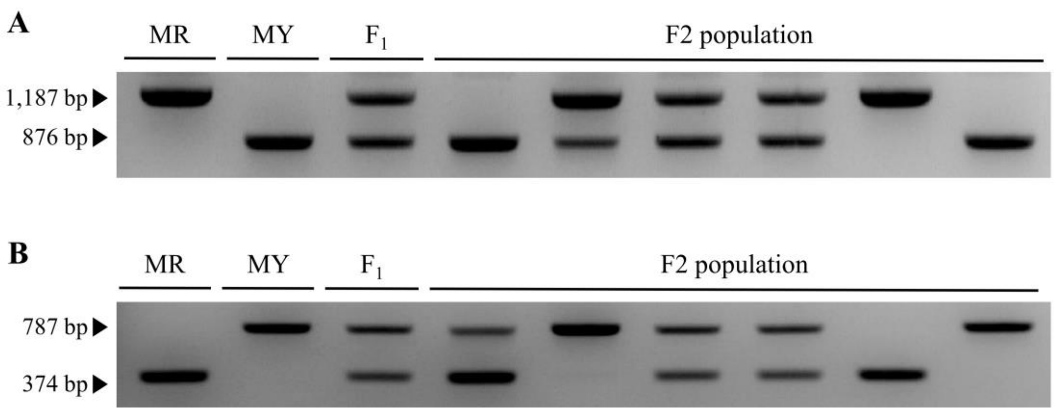

Fig. S3. SCAR marker genotyping of *PSY1* and *CCS*.

```
CaPSY1   MSVALLWVVSPC-DVSNGTGFLVSVREGNRIFDSSGRRN----LACNERIKRGGGKQRWS   55
SlPSY1   MSVALLWVVSPC-DVSNGTSFMESVREGNRFFDSSRHRN----LVSNERINRGGGK----   51
CaPSY2   MSVALLWVVSPNSEVSNGTGFLDSVREGN------RVPGRDRNSMWKERI-KKGGRQRWN   53
SlPSY2   MSVALLWVVSPNSEVSYGTGFLDSVREGNRGLESSRFPSRDRNSMWKGGF-KKGGRQGWN   59
         ***********  :** **.*: ******         .       :  : : **:

CaPSY1   FG--------SCLGGAQTGSGRKFSVRSAIVATPAGEMTMSSERMVYDVVLRQAALVKRQ  107
SlPSY1   ----------------QTNNGRKFSVRSAILATPSGERTMTSEQMVYDVVLRQAALVKRQ   95
CaPSY2   FGSLNAGLRYSDLGGSRTGNGSSFAVQSSLVASPAGEMAVSSEKKVYDVVLKQAALVKRQ  113
SlPSY2   FGFLNADLRYSCLGRSRTENGRSFSVQSSLVASPAGEMAVSSEKKVYEVVLKQAALVKRH  119
                         :* .* .*:*:*:::*:*:** :::**: **:***:*******:

CaPSY1   LRSTDELDVKKDIPIPGTLGLLSEAYDRCSEVCAEYAKTFYLGTMLMTPERRKAIWAIYV  167
SlPSY1   LRSTNELEVKPDIPIPGNLGLLSEAYDRCGEVCAEYAKTFNLGTMLMTPERRRAIWAIYV  155
CaPSY2   LRSTDDLEVKPDIVLPGNLGLLSEAYDRCGEVCAEYAKTFYLGTLLMTPDRRRAIWAIYV  173
SlPSY2   LISTDDIQVKPDIVLPGNLGLLSEAYDRCGEVCAEYAKTFYLGTMLMTPDRRRAIWAIYV  179
         * **::::** ** :**.***********.********** ***:****:**:*******

CaPSY1   WCRRTDELVDGPNASHITPAALDRWEDRLEDVFSGRPFDMLDAALSDTVSKFPVDIQPFR  227
SlPSY1   WCRRTDELVDGPNASYITPAALDRWENRLEDVFNGRPFDMLDGALSDTVSNFPVDIQPFR  215
CaPSY2   WCRRTDELVDGPNASHITPQALDRWEARLEDIFSGRPFDMLDAALSDTVSRFPVDIQPFR  233
SlPSY2   WCRRTDELVDGPNASHITPQALDRWEARLEDIFNGRPFDMLDAALSDTVSRFPVDIQPFR  239
         ***************:*** ****** ****:*.********.*******.*********

CaPSY1   DMIEGMRMDLRKSRYRNFDELYLYCYYVAGTVGLMSVPIMGIAPESKATTESVYNAALAL  287
SlPSY1   DMIEGMRMDLRKSRYKNFDELYLYCYYVAGTVGLMSVPIMGIAPESKATTESVYNAALAL  275
CaPSY2   DMVEGMRMDLWKSRYMNFDELYLYCYYVAGTVGLMSVPIMGIAPESKATTESVYNAALAL  293
SlPSY2   DMVEGMRMDLWKSRYNNFDELYLYCYYVAGTVGLMSVPIMGIAPESKATTESVYNAALAL  299
         **:******* **** ********************************************

CaPSY1   GIANQLTNILRDVGEDARRGRVYLPQDELAQAGLSDEDIFAGRVTDKWRIFMKKQIQRAR  347
SlPSY1   GIANQLTNILRDVGEDARRGRVYLPQDELAQAGLSDEDIFAGRVTDKWRIFMKKQIHRAR  335
CaPSY2   GIANQLTNILRDVGEDARRGRIYLPQDELAQAGLSGEDIFAGRVTDKWRIFMKKQIQRAR  353
SlPSY2   GIANQLTNILRDVGEDARRGRVYLPQDELAQAGLSDEDIFAGKVTDKWRIFMKKQIQRAR  359
         *********************:*************.******:*************:***

CaPSY1   KFFDEAEKGVTELSAASRWPVLASLLLYRRILDEIEANDYNNFTKRAYVSKPKKLIALPI  407
SlPSY1   KFFDEAEKGVTELSSASRFPVWASLVLYRKILDEIEANDYNNFTKRAYVSKSKKLIALPI  395
CaPSY2   KFFDQAEKGVAELSSASRWPVLASLLLYRKILDEIEANDYNNFTRRAYVSKPKKLLTLPI  413
SlPSY2   KFFDEAEKGVTELSSASRWPVLASLLLYRKILDEIEANDYNNFTRRAYVSKPKKLLTLPI  419
         ****:*****:***:***:** ***:***:**************:****** ***::***

CaPSY1   AYAKSLVPSTRT-------                                          419
SlPSY1   AYAKSLVPPTKTASLQR--                                          412
CaPSY2   AYARSLVPPKLTSSSLTKT                                          432
SlPSY2   AYARSLVPPKSTSSPLAKT                                          438
         ***:**** . *
```

Fig. S4. Amino acid sequence alignment of pepper and tomato PSY1 and PSY2.

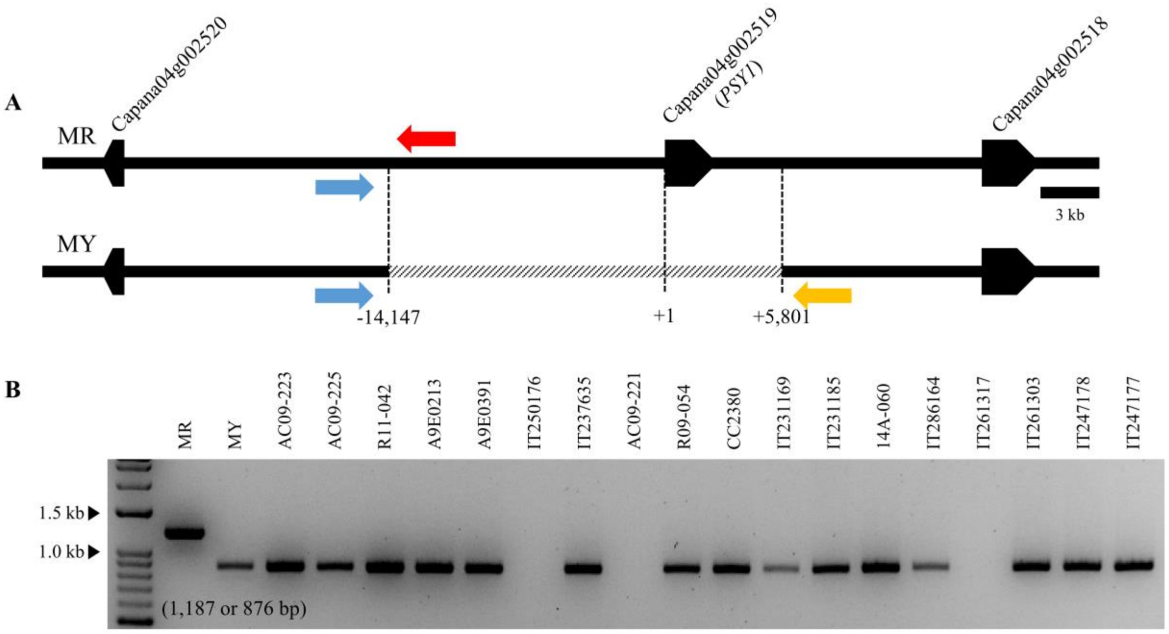

Fig. S5. SCAR marker genotyping of the accessions from which *PSY1* was not previously amplified.

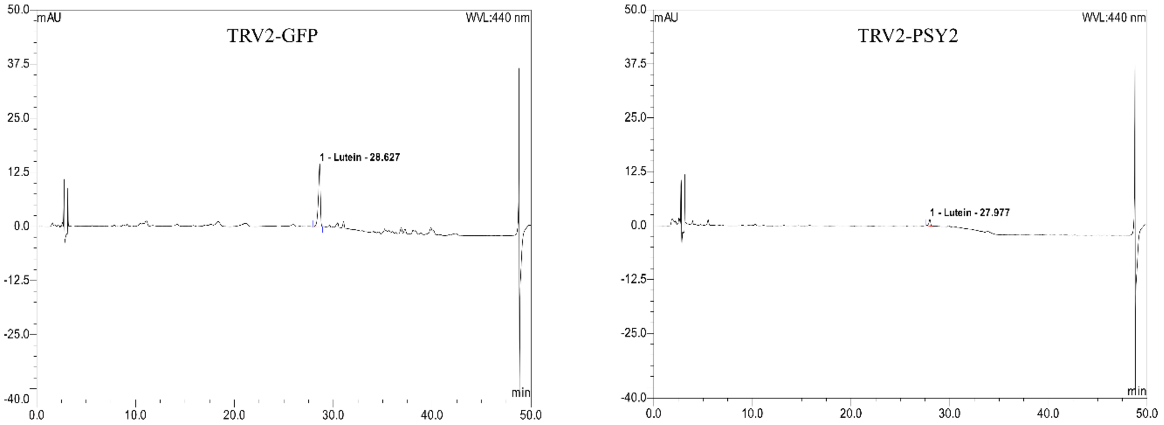

Fig. S6. HPLC profiles of the carotenoid extracts obtained from the VIGS-treated MY plants.

Appendix 1. LTR retrotransposon sequences discovered in the MY *CCS* sequence.

/1–1,333 *CCS* N-terminal; 1,334–1,339 TSD; 1,340–2,381 LTR; 7,164–8,263 LTR; 8,264–8,269 TSD; 8,270–8,427 *CCS* C-terminal

```
    1 ATGGAAACCC TTCTAAAGCC TTTTCCATCT CCTTTACTTT CCATTCCTAC TCCTAACATG
   61 TATAGTTTCA AACACAACTC CACTTTTCCA AATCCAACCA AACAAAAAGA TTCAAGAAAG
  121 TTCCATTATA GAAACAAAAG CAGTACACAT TTTTGTAGCT TTCTTGATTT AGCACCCACA
  181 TCAAAGCCAG AGTCTTTAGA TGTTAACATC TCATGGGTTG ATACTGATCT GGACGGGGCT
  241 GAATTCGACG TGATCATCAT TGGAACTGGC CCTGCCGGGC TTCGGCTAGC TGAACAAGTT
  301 TCTAAATATG GTATTAAGGT ATGTTGCGTT GACCCTTCAC CACTTTCCAT GTGGCCAAAT
  361 AATTATGGTG TTTGGGTTGA TGAGTTTGAA AAGTTGGGAT TAGAAGATTG TCTAGATCAT
  421 AAGTGGCCTG TGAGTTGTGT TCATATAAGT GATCACAAGA CTAAGTATTT GGACAGACCA
  481 TATGGTAGAG TAAGTAGAAA GAAGTTGAAG TTGAAATTGT TGAATAGTTG TGTTGAAAAT
  541 AGAGTGAAGT TTTATAAAGC CAAGGTTTTG AAAGTGAAGC ATGAAGAATT TGAGTCTTCG
  601 ATTGTTTGTG ATGATGGTAG GAAGATAAGC GGTAGCTTGA TTGTTGATGC AAGTGGCTAT
  661 GCTAGTGATT TTATAGAGTA TGACAAGCCA AGAAACCATG GTTATCAAGT TGCTCATGGG
  721 ATTTTAGCAG AAGTTGATAA TCATCCATTT GATTTGGATA AAATGATGCT TATGGATTGG
  781 AGGGATTCTC ATTTAGGTAA TGAGCCATAT CTGAGGGTGA AGAATACTAA AGAACCAACA
  841 TTCTTGTATG CAATGCCATT TGATAGGAAT TTGGTATTCT TGGAAGAGAC TTCTTTAGTG
  901 AGTCGGCCTA TGTTATCGTA TATGGAAGTG AAAAGAAGGA TGGTAGCAAG ATTAAGACAT
  961 TTGGGGATCA AAGTGAGAAG TGTCCTTGAG GAAGAGAAGT GTGTGATCAC TATGGGAGGA
1,021 CCACTTCCGC GGATTCCTCA AAATGTTATG GCTATTGGTG GGACTTCAGG GATAGTTCAT
1,081 CCATCGTCTG GGTACATGGT GGCTCGTAGC ATGGCATTGG CACCAGTACT GGCTGAGGCC
1,141 ATCGTCGAAA GCCTTGGCTC AACAAGAATG ATAAGAGGGT CTCAACTTTA CCATAGAGTT
1,201 TGGAATGGTT TGTGGCCTTC GGATAGAAGA CGTGTTAGAG AATGTTATTG TTTCGGAATG
1,261 GAGACTTTGT TGAAGCTTGA TTTGGAAGGT ACTAGGAGAT TGTTTGATGC TTTCTTTGAT
1,321 GTTGATCCCA AGTACTGGCT GTTGGGTTCA TAGTGTGTTG AATGGAAAAT GGAGAAACCT
1,381 AAGTGAAGGG AAGGAGACAC CCAAATTGGA AAGTGTACTC TTTGCTAAGG AAAGTTTACT
1,441 CTTCTCCCAC ATTGGTGGAA GAAGAGAACT TTAAAGTGTT TAAAAGTGAA ACACTTACTT
1,501 CACATGGTAA GTGAGGCAAG GAAATAGAGA TGCCTCGCGC CGTCGTCGTC GTCGCTCGCT
1,561 CGGCTTGGCT TCGGCTTCGG ATTTGGATTT GGATTTGGTC AAATGATCGA TCGATGAGAT
1,621 CTATTTTTTG GACAAAATTT ATTTGACGAA AATCTGAATA TGTGTAAAAC GCAGGAAATT
```

1,681 GTTTTTTTTC TGTGATTTGG ACAAGGGTTG CAATGATTCC AGATGTAATG TTCTTAAGCA
1,741 GATGCACTGT TTGACGAACA GATGCACTGT TTCATGAATA GATGCTGCTG AAAGTTGCGA
1,801 TTCTTCATGA AACAACACAC TGTTTCATCC GAAATGACAT GCCTGTTCAG AAACAGTCAC
1,861 ACTGGTTGGC CGAATGGACA TGAATTTTCA GAAAAGGTAA CACCTTTTTG ATGAACCTTT
1,921 GTCTCTCTTC GGAAGAGACA TCACTTGGCT ATAAAAACCT GCTTTGATTC ACAGGTTTTA
1,981 ATACAGAATT TTTCAGATTA CAAAACATTC TTCTTGTCTT AAAAATACTC CAAGTGTGAT
2,041 CATACAAACC GTGAGTGTGT TCGAAGAATC TGCCTATTTG AGGTACCGCT ATAGTCGGAT
2,101 TGAAGGCCAT TTTATCCTGG GAGGAAAATT CCACAACCTT GGGTACAGTG AGGGGAATTA
2,161 TTTCCTTAAG GAAAGTCCGT GAATTCGGAT GACTTGGCCT TAACAACATT TCTGTTTCAT
2,221 CTAATTTTTC TGAAAAACAC ACTTCTTTGA AAGATCTATT TTGATCTTGT GTTGAGGGTG
2,281 TTACTTAACT TCATAACGTT CTTGTTTTGT ACTTGACCTA GCGTTGAAGT TGTGTTTCTA
2,341 TATTATACAG ATTGTACGTA CCCGTATCGG CGGTGGATAA CAATCTTAAG GAAATAATCC
2,401 TTGGAATCTG TATAACTTGT TTTTGGAGAT TAAAGCCTAT GCTTACTACT CCGTTTGAAT
2,461 TTAACTTGTG ATTAGAAGAC ATAAAATCTT CATCAAAAGT TGAAGTTCAC TCGATTGACG
2,521 ATTGGAAGTT ATAAAAACTT CGTCGTTAAT TAGAAACAAG AAGGAAAAAC TTATAAAGTT
2,581 ATTTAACTTT ATTAATAGTA TTCTGGAAAA TACTAATTTT CTGTCTTGTG GTGACAGGAA
2,641 AAATGACAAC TGAAGGTCAA ATTATGAGTG CAACAACGTC GGTGGGGGCA ACTAACATTG
2,701 CCACATCTAG TCGCACAACT GCTCCGCCAT CGATGGCCCC GGCGGAGAAG CCCGGAAAAT
2,761 TTTCTGGCAT CGACTTCAAA CGGTGGCAAC AAAAGATGTT CTTTTATCTC ACCACGTTAT
2,821 GTCTACAAAG GTTCACTAGT GAAGATGCAC CCGAGGTACC CGAGGGAACC TCGGACAAGG
2,881 ATCGATTCGT CATTATAGAA GCTTGGAAAC ATTCAGATTT CCTTTGCAGG AACTACATTC
2,941 TGAGCGGTCT CCAAGATGAT CTCTATAATG TCTATAGTGG GACCAAGACA GCAAAAGAAC
3,001 TGTGGGAGGC GCTTGAACGG AAATATAAAA CGGAAGATGC GGGAATTAAG AAATTCCTTG
3,061 TTGCACGATT CCTGGACTTT AAAATGATTG ATAGCAAATC TGTTGTCTCT CAAGTACAGG
3,121 AGTTGCAAGT CATCATACAT GATCTCCTAG CAGAAGGTTT AATTGTGAAT GATGCTTTTC
3,181 AAGTAGCAGC AATGGTTGAG AAGCTACCAC CTTTGTGGAA AGACTTCAAA AACTACTTAA
3,241 AGCATAAGCG CAAGGAGATG ACCGTTGAAG ATCTTATTGT TCGACTTCGG ATTGAAGAAG
3,301 ACAATAAAGC TGCCGAAAGA AGGTCAAAGG GAAATTCTGC AATGAATGGA GCACATATTG
3,361 TAGAAGATGG CCAAAACAAC TCGAAGAAAA GAAAGAATGT TGAACATGGA AGCAATCATC
3,421 CCAAGAAAAA GTTTAAGGGA AAGTGCTTCA ACTGTGGCAA AGTTGGCCAC AAATCAACAG
3,481 ACTGTCGAGC CTCGAAAAAA GGGAAGAAAA AGGACCAAGC AAATCTGATT GAGTCCAACA

3,541 AGGATTATGA CGACCTGTGT GCTATGTTCA CGGAATGCAA CATGGTCGGC AATCCACGTG
3,601 CTTGGTGGAT GGATTCAGGG GCCACACGCC ATGTCTGCGC AAGCAAAGAG TTGTTCTCGA
3,661 CATATGCTCC GGCTCAAGCG GAAGAAACAA TATACATGGC CAACTCCGCG ACTGCTAAGG
3,721 TGGAGGGAAT AGGAAAAGTT TGCCTAAAGA TGACATCTGG AAAGGTTTTG ACACTAAATA
3,781 ATGTGTTATA TGTTCCGGAG TTACGTAGGA ACTTAATTTC TGTTTCACTC CTAGATAAAA
3,841 ATGGTTTCAA ATGTGTAACC GTTTCTGGAA AAATAGTAGT TAGCAAAGGA GAAATGTATG
3,901 TAGGAAAAGG CTATCTCGAG GAAGGCCTTT ATAAGATGAA TGTAATGAAT ATTGAAATGA
3,961 ATAAAAATTC AAATTCTTCT TACTTGCTTG AGTCTTATAA TTTATGGCAT GAACGTTTAG
4,021 GCCATGTTAA TTACAAAACG TTACGAAAAC TGATTAACTT AGAAGTTTTG CCAAATTGTG
4,081 AGTGCAATAA ATCTAAGTGT CAAACGTGTG TAGAATCAAA GTATGCTAAG CATCCTTATA
4,141 AGTCTGTTGA AAGGAATTCC AATCCCTTAG ACTTAATACA CACTGACATT TGTGATATGA
4,201 AGTCAACACC ATCACGTGGT GGAAAAAAGT ATTTCATAAC TTTTATTGAC GATTGCACTA
4,261 GATATTGTTA TGTCTACTTG CTAAATAGTA AGGATGAAGC AATAGATGCG TTTAGGCAAT
4,321 ATAAAACTGA AGTTGAAAAT CAGTTAGACA AAAGGATCAA AATGATAAGA AGTGACAGGG
4,381 GCGGAGAATA TGAATCTCCC TTTGCGCAAA ATATGTGTAG AGAATGGAAT AATCCATCAA
4,441 ACTACGGCCC CCGTATTCAC CTCAATCTAA TGGAATTGCT GAAAGGAAAA ATCGAACTTT
4,501 GAAGGAAGTG ATGAATGCCT TACTAATAAG TTCTGGTTTA CCGCAAAACT TGTGGGGGGA
4,561 GGCTATCCTT ACGGCCAATC GTATACTCAA TAGAGTTCCC CATAGTAAGA CACAATCAAT
4,621 TCCTTACGAA AAATGGAAAG GAAGGAAACC CAACTTGAAA TATTTCAAAG TGTGGGGGTG
4,681 TCTAGCGAAG GTTCAAGTTC CTATATCTAA AAGGGTTAAG ATAGGACCTA AAACGGTGGA
4,741 CTGCGTGTTC ATAGGATATG CTAAAAGGAG TAAAGCATGT CGATTTTTGG TTCATAAATC
4,801 CGAACATTCG GATATAAATG AAAATACGGT AATTGAATCA GATAATGCTG AATTCTTTGA
4,861 AAACATTTAT CCGTATAAAA CTAGACATGA ACAGTCTAGT GGAGGATCTA AACGACCCCG
4,921 AGATGAACCA AGTGAGAATG TACATAATGA AGAAAATCCA AGACGTAGTA CACGTCAAAG
4,981 AACATCAACT TCGTTTGGAT CGGATTTTGT AACGTTTCTC TTAGAAAATG AGCCTCGAAC
5,041 GTTTAAAGAA GCGATGTCGT CATCAGACTC ATCCTTTTGG AAAGAGGCAG TCAATAGTGA
5,101 GATTGATTCA ATCTTAAGTA ACCATACGTG GGAATTAGTT GATCTTCCTC CAGGAAATAA
5,161 ACCTTTAGGG TCTAAATGGA TCTTCAAAAG GAAAATGAAA ACGGATGGTT CTATTGACAA
5,221 ATACAAGGCA AGACTTGTAG TAAAAGGCTT CAATCAAAAA GAAGGTCTTG ATTACTTTGA
5,281 TACATACTCG CCAGTGACAA GGATAACCTC AATTCGAATG TTAATTGCCT TGGCGGCAGT
5,341 ATATAATCTT CAAATCCATC AAATGGATGT GAAAACCGCA TTCCTAAACG GAGAATTGGA

5,401 GGAAGAAATC TACATGGAAC AACCTGAGGG TTTTGTGGTT CCAGGAAAAG AAAATAAGGT
5,461 GTGTAAACTT GTTAAGTCAC TTTATGGACT AAAACAAGCA CCCAAGCAAT GGCATGCAAA
5,521 GTTTGACCAA ACCATGTTGG CAAACGGATT CAAGATAAAT GAATGTGATA AATGTGTGTA
5,581 TATTAAGGAC ACACCAAATC ACCAAGTCAT TGTATGTTTA TATGTGGATG ATATGTTGAT
5,641 CATCAGTAGA GACATTTGTG ACATAAATGC AACCAAACAA ATGCTCGAGA GCAAGTTTGA
5,701 TATGAAAGAC CTCGGAGTTG CAGATGTGAT CTTAGGTATA AGAATCCATC GAACTCTACA
5,761 AGGGTTAGCA CTGTCACAGT CTCATTATAT CGAAAAAGTA CTTGACAAGT TCAAGTATAT
5,821 GGAGTTCGAT ATTGCCAAGA CTCCATTGGA TGCGAACTTT GCACTTCGGA AAAATGAAGG
5,881 TGAAAGTGAT TCTCAATTGG AGTACGCAAG AGTATTGGGA TGTTTAATGT ATATAATGAA
5,941 CTGTACACGA CCAGACATAG CATGCACTAT CAGTAAATTG AGTCGGTACA CGAGTAATCC
6,001 CAATAAAACT CACTGGATGG CAGTGAAAAG AGTTTTGGGT TATCTTAAAT ACACTCGAGA
6,061 CTATGCTTTG CATTATAATA AATATCCAGC GGTACTTGAA GGATATAGTG ATGCAAATTG
6,121 GATCACCGGA TCGAACGAAG TAAAATCCAC AAGTGGATAT GTATTTACTA TCGGTGGAGG
6,181 AGCAGTTTCT TGGAAATCGT CGAAACAGAC TTGTATCGCT CGCTCTACAA TGGAATCTGA
6,241 ATTTATCGCA TTAGATAAAG CCGGTGAAGA AGCAGAATGG CTCCGGAATT TCTTAGAAGA
6,301 TATTCCTTAT TGGCCCAAGC CAGTGGCACC AGTATGTATA CACTGTGATA GCCAAGCGGC
6,361 AATAGGTAGG GCTGGGAGCA TGATGTACAA CGGTAAGTCT CGTCACATAC GTCGAAGACA
6,421 TAATACCGTT AGAGAACTTC TCTCTAGTGG AATTATTACT ATAGACTATG TAAAGTCAAA
6,481 GGATAATGTG TCGGATCCAC TTACAAAAGG CCTATCTAGA GAAGGAGTAG AAAGGACATC
6,541 CAAGGGAATG GGTTTAAGGC CTAGGACAAG TCAGCATGGC GGTAACTCTA CCTAGCAGAC
6,601 TGGAGATCCC AAGAGCTAGG TTCAAGGAGA ACAAACAAAG TTATGTCTGA CAGGTTCAAC
6,661 ATTGTCATTC ACCCAACTCA TTCTCATGAT GTAGACAATG TATAGTAAAC CAGGATAAGA
6,721 CTTAAGGTGA AAAGTCTTTT AATGATTATC TAAATTTGGC AGATTTGACC AAATAGTTCA
6,781 AGCTAAAGGA TTGAAACGTT TAGAAATCAC CTATATGAGG GCGAAGTGGA AGCCGCTTCA
6,841 AAGAGAATGT TAGTAAAGGC CTATTCTCTA AGCTCTCATG AAACCGGGAC GTGTTCATGG
6,901 CTGAAAAGAA CAAAACCGTA AGAACCATAA ATGGTAAAAG GCTGATTGTG TGACATGTGT
6,961 TGTCTAGGTG TACATTAAAG CTCGACGGTT CAAAGATATC AAATCTACCG ATTGACCGAG
7,021 TGCATCCGAT ACATGTTCAC TACGGAAAGT TCAAAGGGAA ACCCACTTAT CCAGATGCAA
7,081 TCAGTCCTTG CTTGATGATC ACATACTGTC CGTAAACGAT TTTTGAAAAA AAAAAATAGC
7,141 CATTCCCCAT TCATGTGGGG GATTGTTGGG TTCATAGTGT GTTGAATGGA AAATGGAGAA
7,201 ACCTAAGTGA AGGGAAGGAG ACACCCAAAT TGGAAAGTGT ACTCTTTGCT AAGGAAAGTT

7,261 TACTCTTCTC CCACATTGGT GGAAGAAGAG AACTTTAAAG TGTTTAAAAG TGAAACACTT
7,321 ACTTCACATG GTAAGTGAGG CAAGGAAATA GAGATGCCTC GCGCCGTCGT CGTCGTCGCT
7,381 CGCTCGGCTT GGCTTCGGCT TCGGCTTCGG CTTTGGATTT GGATTTGGAT TTGGATTTGG
7,441 TCAAATGATC GATCGATGAG ATCTATTTTT TGGACAAAAT TTATTTGACG AAAATCTGAA
7,501 TATGTGTAAA ACGCAGGAAA TTGTTTTTTT TCTGTGATTT GGACTCCATT TATATGCATG
7,561 CAGTGAACTG CTGCAGAAAC GTTAAAGGGT TGCAATGATT CCAGATGTAA TGTTCTTAAG
7,621 CAGATGCACT GTTTGACGAA CAGATGCACT GTTTCATGAA TAGATGCTGC TGAAAGTTGC
7,681 GATTCTTCAT GAAACAACAC ACTGTTTCAT CCGAAATGAC ATGCCTGTTC AGAAACAGTC
7,741 ACACTGGTTG GCCGAATGGA CATGAATTTT CAGAAAAGGT AACACCTTTT TGATGAACCT
7,801 TTGTCTCTCT TCGGAAGAGA CATCACTTGG CTATAAAAAC CTGCTTTGAT TCACAGGTTT
7,861 TAATACAGAA TTTTTCAGAT TACAAAACAT TCTTCTTGTC TTAAAAATAC TCCAAGTGTG
7,921 ATCATACAAA CCGTGAGTGT GTTCGAAGAA TCTGCCTATT TGAGGTACCG CTATAGTCGG
7,981 ATTGAAGGCC ATTTTATCCT GGGAGGAAAA TTCCACAACC TTGGGTACAG TGAGGGGAAT
8,041 TATTTCCTTA AGGAAAGTCC GTGAATTCGG ATGACTTGGC CTTAACAACA TTTCTGTTTC
8,101 ATCTAATTTT TCTGAAAAAC ACACTTCTTT GAAAGATCTA TTTTGATCTT GTGTTGAGGG
8,161 TGTTACTTAA CTTCATAACG TTCTTGTTTT GTACTTGACC TAGCGTTGAA GTTGTGTTTC
8,221 TATATTATAC AGATTGTACG TACCCGTATC GGCGGTGGAT AACACTGGCA CGGGTTCCTT
8,281 TCTTCAAGAT TGTCTGTCAA AGAACTTGCT GTACTCAGTT TGTACCTTTT TGGACATGCC
8,341 TCTAATTTGG CTAGGTTGGA TATTGTTACA AAGTGCACTG TCCCCTTGGT TAAACTGCTG
8,401 GGCAATCTAG CAATAGAGAG CCTTTGANNN
